# Preventing acute gut wall damage in infectious diarrhoeas with glycosylated dendrimers

**DOI:** 10.1002/emmm.201201290

**Published:** 2012-08-06

**Authors:** Ian Teo, Steve M Toms, Benoit Marteyn, Teresa S Barata, Peter Simpson, Karen A Johnston, Pamela Schnupf, Andrea Puhar, Tracey Bell, Chris Tang, Mire Zloh, Steve Matthews, Phillip M Rendle, Philippe J Sansonetti, Sunil Shaunak

**Affiliations:** 1 Departments of Medicine, Infectious Diseases & Immunity, Imperial College London, Hammersmith HospitalDucane Road, London, UK; 2 Industrial Research (IRL)Lower Hutt, New Zealand; 3 Institut Pasteur, Unité de Pathogénie Microbienne Moléculaire & Unité INSERM 786Paris, France; 4 UCL School of Pharmacy, University College LondonLondon, UK; 5 Cross-Faculty NMR Centre & Department of Life Sciences, Imperial College LondonSouth Kensington, London, UK; 6 Sir William Dunn School of PathologySouth Parks Road, Oxford, UK

**Keywords:** dendrimer, diarrhoea, infection, interleukin-6, nanobiotechnology

## Abstract

Intestinal pathogens use the host's excessive inflammatory cytokine response, designed to eliminate dangerous bacteria, to disrupt epithelial gut wall integrity and promote their tissue invasion. We sought to develop a non-antibiotic-based approach to prevent this injury. Molecular docking studies suggested that glycosylated dendrimers block the TLR4-MD-2-LPS complex, and a 13.6 kDa polyamidoamine (PAMAM) dendrimer glucosamine (DG) reduced the induction of human monocyte interleukin (IL)-6 by Gram-negative bacteria. In a rabbit model of shigellosis, PAMAM-DG prevented epithelial gut wall damage and intestinal villous destruction, reduced local IL-6 and IL-8 expression, and minimized bacterial invasion. Computational modelling studies identified a 3.3 kDa polypropyletherimine (PETIM)-DG as the smallest likely bioactive molecule. In human monocytes, high purity PETIM-DG potently inhibited *Shigella* Lipid A-induced IL-6 expression. In rabbits, PETIM-DG prevented *Shigella*-induced epithelial gut wall damage, reduced local IL-6 and IL-8 expression, and minimized bacterial invasion. There was no change in β-defensin, IL-10, interferon-β, transforming growth factor-β, CD3 or FoxP3 expression. Small and orally delivered DG could be useful for preventing gut wall tissue damage in a wide spectrum of infectious diarrhoeal diseases.

–>See accompanying article http://dx.doi.org/10.1002/emmm.201201668

→ See accompanying article http://dx.doi.org/10.1002/emmm.201201668

## INTRODUCTION

Infection with just 100 *Shigella* bacilli is sufficient to cause a severe inflammatory bloody diarrhoea that affects 165 million people worldwide and leads to >1 million deaths annually (Kosek et al, 2010). Vaccine development is impaired by the antigenic diversity of the O-antigen of *Shigella* lipopolysaccharide (LPS), and antibiotics do not prevent the pathogen-induced and cytokine-mediated tissue injury. This has led to increasing interest in modulating the host's intestinal immune response to enteric pathogens (Arpia et al, 2011; Bruckner & Finlay, 2011), which is characterized by an early cytokine-mediated inflammatory response (Abreu, 2010; Ashida et al, 2011). The pathogen-induced tissue injury is mediated, in part, by the TLR4-MD-2-LPS complex (Ohto et al, 2007, 2012; Park et al, 2009), and shigellosis provides a well-defined *in vivo* model for studying such damage (Perdomo et al, 1994; Raqib et al, 1995; Schnupf & Sansonetti, 2012; Singer & Sansonetti, 2004). Both *Shigella* and *Salmonella* can also activate Type III secretory systems (TTSS) to enhance their gut wall invasion (Konradt et al, 2011). A prompt macrophage TLR4-MD-2-LPS complex-mediated cytokine response destroys pathogenic bacteria even if the bystander cost is severe host organ tissue damage.

Dendrimer nanotechnologies use highly controlled and sequential processes to make branched symmetrical molecules (Hourani & Kakkar, 2010; Menjoge et al, 2010). Anionic dendrimers have physico-chemical properties that are similar to small molecule drugs, exist at physiological pH as zwitterions, are not toxic or immunogenic, and preferentially accumulate in tissues containing inflammatory cells (Kannan et al, 2012; Malik et al, 2000). Previous studies showed that a combination of dendrimer glucosamine (DG) and dendrimer glucosamine 6-sulphate prevented scar tissue formation (Shaunak et al, 2004). Molecular modelling studies also suggested that partial surface glycosylation of divergently synthesized anionic dendrimers confers physico-chemical properties that enable co-operative electrostatic interactions with MD-2 in the TLR4-MD-2-LPS complex (Barata et al, 2011a, b).

The additional finding that azabisphosphonate-capped dendrimers selectively target monocytes and can also suppress pro-inflammatory cytokines in mice with inflammatory arthritis (Hayder et al, 2011) led us to translate our mechanistic observations into a new dendrimer-based molecule. Our aim was to create chemically well-defined molecules that would be highly bioactive against the cytokine-mediated epithelial gut wall damage that occurs in a wide spectrum of inflammatory intestinal pathogen-mediated diarrhoeas (Vaisman et al, 2003). Our plan for achieving this aim was to: (i) obtain *in vitro* and *in vivo* biological proof-of-principle data with a large and commercially available polyamidoamine (PAMAM) dendrimer that we could glycosylate; (ii) identify and make a novel, much smaller and simpler glycosylated dendrimer that had better *in vitro* and *in vivo* bioactivity over a longer period of time.

## RESULTS

### *In vitro* studies of PAMAM-DG with LPS and *E. coli*

We started with an UPLC (ultraperformance liquid chromatography)-purified 11.5 kDa diaminobutane core PAMAM dendrimer (PAMAM-D) with 64 peripheral carboxylic acids to which nine surface glucosamine molecules were covalently conjugated (Shaunak et al, 2004 and Supporting Information Fig S1A and B). PAMAM-DG had no antibacterial activity at 5 mg/ml against the Gram-negative Enterobacteriaceae *Escherichia coli* (Supporting Information Fig S2). We used *E. coli* for some of the initial experiments because *Shigella* belongs to the *E. coli* species, and its Lipid A (a 1,4′-bis-phosphorylated diglucosamine backbone to which variable lengths and numbers of acyl chains are covalently linked that anchors LPS to the outer membrane of Gram-negative bacteria) is identical to that of *Shigella* (Lindberg et al, 1991).

**Figure 1:**
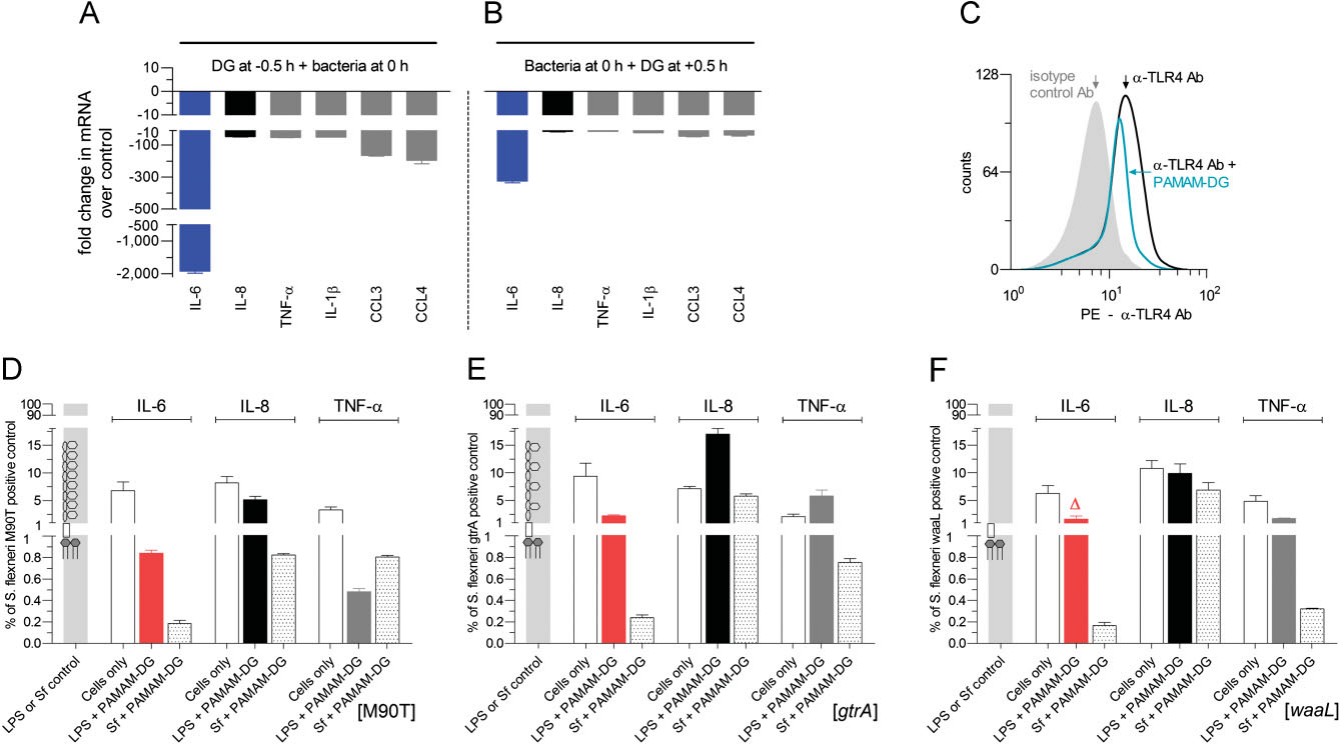
*In vitro* cellular bioactivity of PAMAM-DG **A.** IL-6, IL-8 (CXCL-8), TNF-α, IL-1β, CCL3 (MIP-1α) and CCL4 (MIP-1β) mRNA levels were reduced (*p* < 0.0001) when PAMAM-DG (200 µg/ml) was added to human monocytes 30 min before *E. coli*. Pooled data (*n* = 9) shown as mean ± sem.**B.** IL-6, IL-8, TNF-α, IL-1β, CCL3 and CCL4 mRNA levels were reduced (*p* < 0.0001) when *E. coli* were added to human monocytes 30 min before PAMAM-DG (200 µg/ml). Pooled data (*n* = 9) shown as mean ± sem.**D.** Preincubation of human monocytes with PAMAM-DG (200 µg/ml) for 1 h reduced anti-TLR4 antibody binding by 35 ± 7%. Pooled data: *n* = 8.**D–F.** Preincubation of human monocytes with PAMAM-DG (200 µg/ml) for 1 h inhibited the induction of IL-6, IL-8 and TNF-α in human monocytes by: (i) ultrapure *Shigella* LPS and wild type *S. flexneri* (Sf; M90T strain) (**D**); (ii) ultrapure LPS-*gtrA* (shorter O-chains) and the genetically engineered strain *gtrA* (**E**); (iii) ultrapure LPS-*waaL* (endotoxin component of LPS that binds only to MD-2) and the genetically engineered strain *waaL* (**F**). Δ = 1.66 ± 0.55%. Pooled data (*n* = 3) shown as mean ± sem. *p* < 0.0001; *p* values determined using a two-tailed Mann–Whitney test.

When endotoxin free [<0.06 endotoxin units (EU)/ml, which is the EU standard for water for injection] PAMAM-DG was added to primary human monocytes before adding *E. coli* at a multiplicity of infection of 5, there was a 1923-fold reduction in interleukin (IL)-6 messenger RNA (mRNA) expression with a mean reduction of 103 ± 32-fold in IL-8 (CXCL-8), tumour-necrosis factor (TNF)-α, IL-1β, chemokine (C-C motif) ligand 3 (CCL3) (MIP-1α) and chemokine (C-C motif) ligand 4 (CCL4) (MIP-1β) mRNA expression (Fig 1A). The mRNA ED_50_ (PAMAM-DG concentration that reduced mRNA expression by 50% compared to the bacterial positive control) was 200 ± 15 µg/ml (15 µM) and the protein ED_50_ was 20 ± 2 µg/ml (1.5 µM). When bacteria were added to primary human monocytes 30 min before the PAMAM-DG, a 325-fold reduction in IL-6 expression with a mean reduction of 27 ± 6-fold in IL-8, TNF-α, IL-1β, CCL3 and CCL4 expression was still seen (Fig 1B). These results show that PAMAM-DG inhibited *E. coli*-induced cytokine responses in human monocytes.

Polyamidoamine-dendrimer glucosamine competed partially with an anti-TLR4 antibody (HTA125) on human monocytes (Fig 1C). We had previously shown that there was reversibility of the antagonist activity of PAMAM-DG on the induction of human monocyte cytokines by LPS and that glucosamine and the PAMAM dendrimer, when used alone, had no effect on LPS-induced cytokine responses (Shaunak et al, 2004). PAMAM dendrimer glucosamine 6-sulphate and PAMAM dendrimer glucosamine 6-phosphate were also synthesized by conjugating glucosamine 6-sulphate and glucosamine 6-phosphate, respectively, to the PAMAM dendrimer using *N*-(3-dimethylaminopropyl)-*N*′-ethylcarbodiimide hydrochloride (EDCI) and the conjugation chemistry previously described (Shaunak et al, 2004; Sam et al, 2010). Neither the sulphated nor the phosphorylated PAMAM-DG had any effect on LPS-induced cytokine responses in human monocytes (Supporting Information Fig S3).

### *In vitro* studies of PAMAM-DG with LPS and *Shigella*

Polyamidoamine-dendrimer glucosamine had no antibacterial activity against the Gram-negative Enterobacteriaceae *Shigella flexneri* at 7 mg/ml (Supporting Information Fig S4). The relative contribution of the O-antigen and Lipid A to PAMAM-DG's bioactivity was determined by constructing genetically engineered strains of *S. flexneri* that express structurally defined modifications of LPS; *gtrA* has shorter O-chains and *waaL* expresses an endotoxin component of LPS that binds only to MD-2 (Molinaro et al, 2008; Rallabhandi et al, 2008; West et al, 2005). Endotoxin-free PAMAM-DG inhibited the induction of IL-6, IL-8 and TNF-α in human monocytes by: (i) ultrapure *Shigella* LPS and by wild type *S. flexneri* (M90T strain at a multiplicity of infection of 10; Fig 1D); (ii) ultrapure LPS-*gtrA* and the genetically engineered strain *gtrA* (Fig 1E); (iii) ultrapure LPS-*waaL* and the genetically engineered strain *waaL* (Fig 1F). The percentage inhibition of LPS-induced IL-6, IL-8 and TNF-α in human monocytes by PAMAM-DG ranged from 80–99.8% and it was greatest for IL-6 at 97.5–99.8%. Taken together, these results suggested that DG was competing with the Lipid A moiety of LPS for MD-2 rather than with the oligosaccharides of LPS.

### Reductionist molecular modelling studies

**Figure 2:**
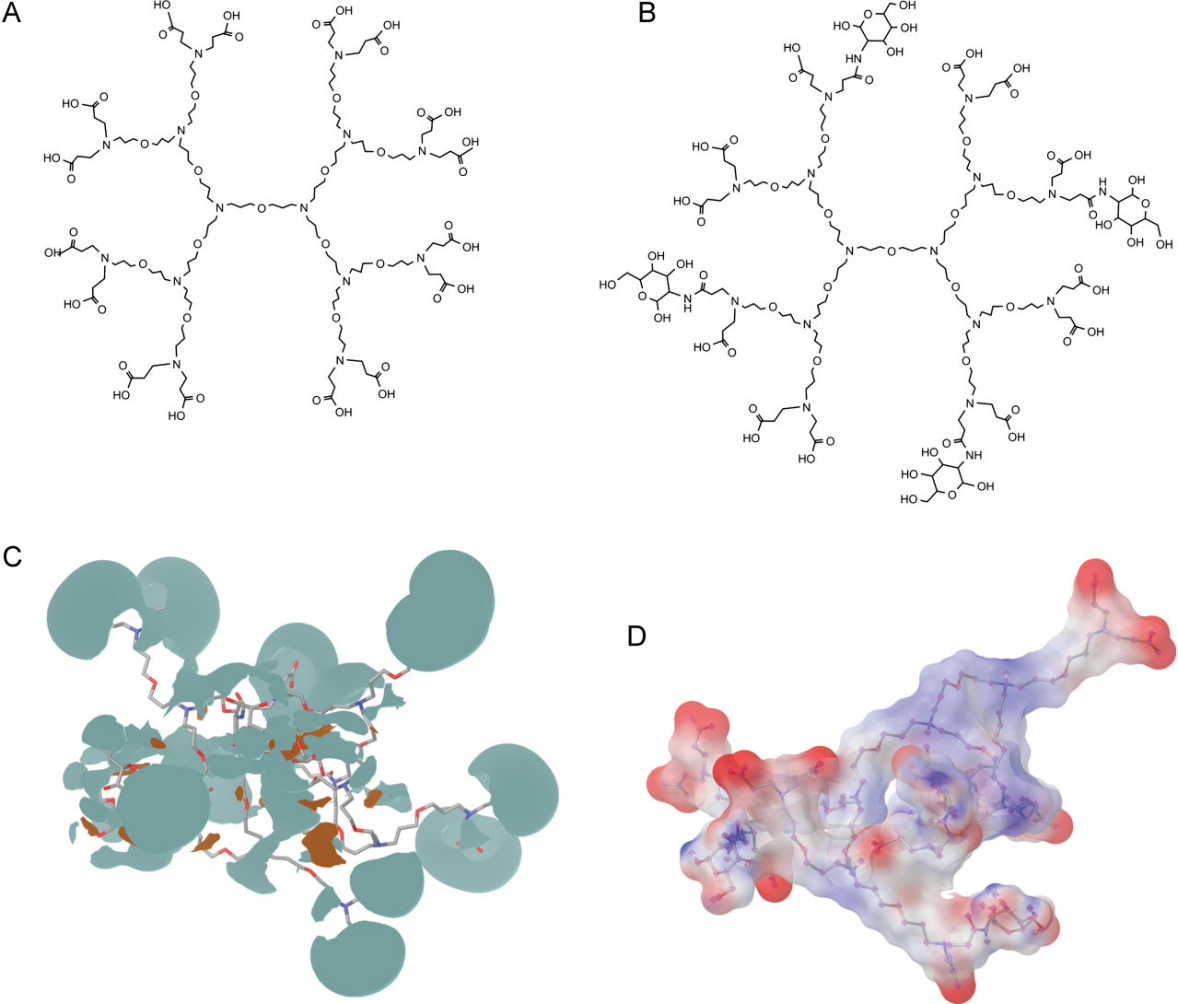
Structures for PETIM-(COOH)_16_ and PETIM-DG Anionic PETIM-(COOH)_16_ dendrimer.PETIM-DG.Hydrophilic (pale blue) and hydrophobic (brown) surfaces of PETIM-DG.Charge surfaces for PETIM-DG with four surface glucosamines; red is negative surface charge and blue is positive surface charge.

The large size (13.6 kDa) of the divergently synthesized and glycosylated PAMAM dendrimer made it difficult to affordably scale-up its synthesis and development. A search was therefore undertaken for a next generation and divergently synthesized dendrimer that was a smaller and simpler dendritic structure using a molecular modelling-based approach and our recently described design principles (Barata et al, 2011c). A combination of modelling and experimental studies suggested that PAMAM-DG's flexibility, surface glucosamine cluster density, electrostatic charge and hydrophilicity could be reproduced in an anionic PETIM dendrimer (Fig 2A) with just 12 peripheral carboxylic acids and four surface glucosamine molecules (MWt 3311 Da; Fig 2B). Molecular dynamics simulations demonstrated that this PETIM-DG exhibited the same flexibility as PAMAM-DG with root mean square deviations for atomic positions of 10 and 11 Å, respectively. Its hydrophilic surface (Fig 2C) and interpolated surface charges [neutral with areas of well separated negative charges (Fig 2D)] closely resembled PAMAM-DG.

Moreover, (i) the human MD-2 binding sites of PAMAM-DG and PETIM-DG shared a common three residue interface (Human/rabbit MD-2: R96K;Y102Y;S118P), and (ii) both PAMAM-DG and PETIM-DG interfered with: (i) the primary electrostatic binding of the 4′-phosphate on the diglucosamine of the Lipid A of LPS to Ser^118^ on MD-2, and (ii) with the secondary binding of TLR4 to Tyr^102^ on MD-2 (Barata et al, 2011b). These molecular modelling observations provided an explanation for the bioactivity of DG. They were also consistent with the recent finding that re-orientation of the aromatic side chain of Phe^126^ in MD-2 is initiated by the binding of the fatty acyl chains of the Lipid A of LPS to MD-2's hydrophobic cavity, and that this is followed by the binding of the MD-2-LPS complex to TLR4 and TLR4-MD-2-LPS receptor complex formation (Yu et al, 2012).

### PETIM dendrimer synthetic studies

**Figure 3:**
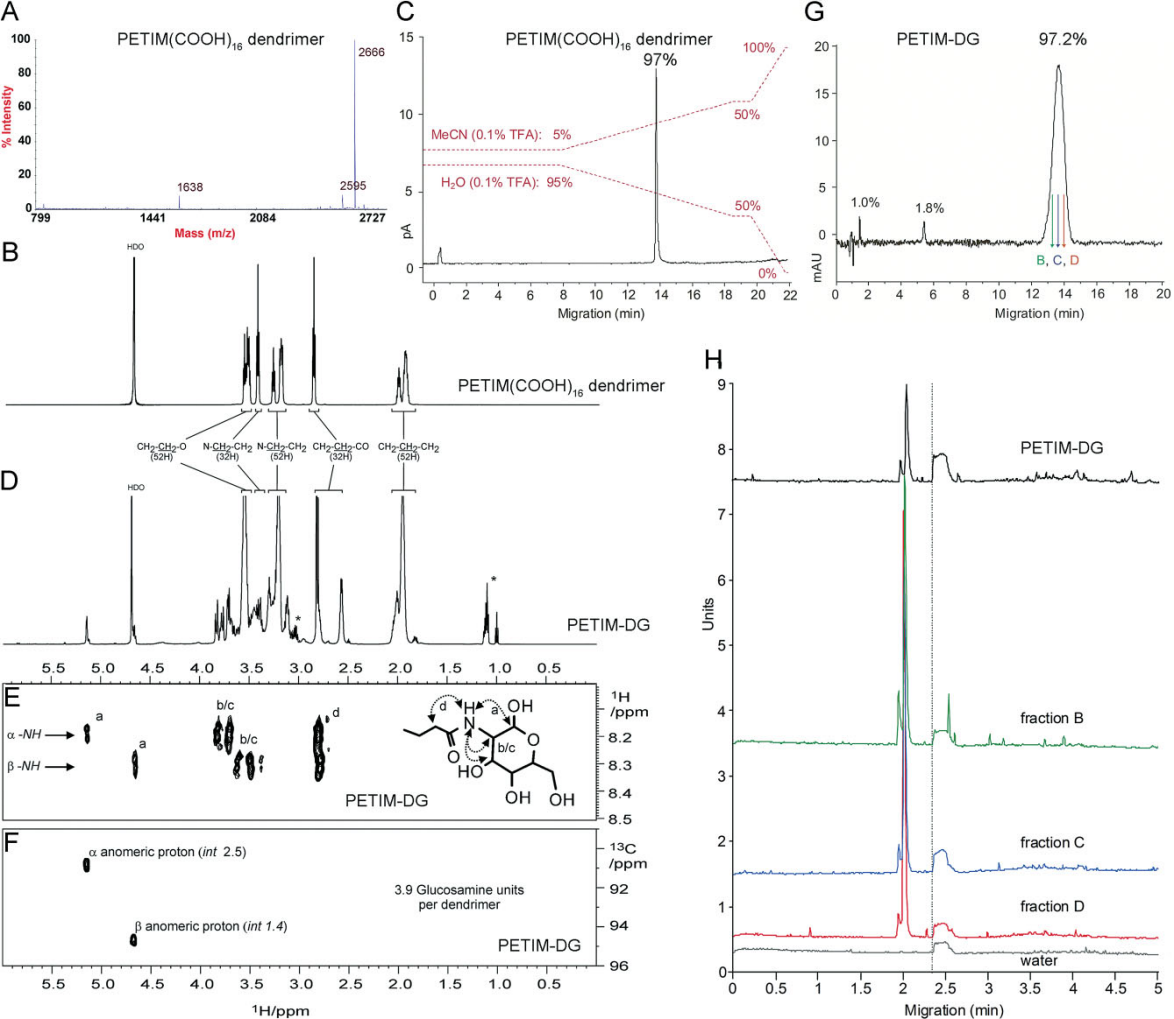
MS, NMR, HPLC and CE chemical analytics for PETIM-(COOH)_16_ and PETIM-DG MALDI-TOF-MS of PETIM-(COOH)_16_ dendrimer (M + H)^+^ = 2666 Da.1D ^1^H NMR (100% D_2_O) of PETIM-(COOH)_16_ dendrimer.HPLC-CAD of PETIM-(COOH)_16_ dendrimer.1D ^1^H NMR (100% D_2_O) of PETIM-DG. *Residual *N*-acylurea and *N*-(3-dimethylaminopropyl)-*N*′-ethylurea (EDU). See also Supporting Information Fig S5.Amide region from 2D ^1^H–^1^H ROESY (rotating-frame Overhauser effect spectroscopy; 90:10 H_2_O:D_2_O) of PETIM-DG showing intermolecular ROE peaks from amide proton to anomeric proton, proximal sugar ring protons, and across the peptide bond (NH–CO–CH_2_).α and β anomeric protons of the C1 carbon of glucosamine from 2D ^1^H–^13^C HSQC (heteronuclear single quantum correlation) NMR (100% D_2_O). Integrals are relative to resolved peaks for 52 CH_2_–CH_2_–CH_2_ protons.HPLC-UV of PETIM-DG. See also Supporting Information Figs S6–S8.CE (capillary electrophoresis) at pH 8.55 of PETIM-DG, and of 3 of 11 fractions collected and analyzed from the HPLC peak shown in (**G**). The main PETIM-DG peak and the main peak in all of the three fractions shown in (**G**) (fractions B, C and D) are aligned at 2.00 min; the water peak in each sample was used to align all four CE chromatograms.

Polypropyletherimine-(COOH)_16_ dendrimer containing ether linkages, tertiary amine branching sites and *n*-propyl spacer groups was chemically synthesized at the 15 g scale with divergent dendrimer growth occurring through iterative Michael additions and functional group reductions (Jayamurugan & Jayaraman, 2006; Krishna and Jayaraman, 2003). The purity of the reagents used and of the alcohol, nitrile, amine and ester intermediates formed was >95% as determined by high performance liquid chromatography-charged aerosol detection (HPLC-CAD). PETIM-(COOH)_16_ dendritic identity was confirmed by matrix-assisted laser desorption/ionization–time-of-flight mass spectrometry (MALDI-TOF-MS; MWt = 2665 Da; Fig 3A) and NMR (nuclear magnetic resonance) spectroscopy (Fig 3B). Purity by HPLC-CAD was 97% (Fig 3C). PETIM-(COOH)_16_ dendrimer has been shown previously not to be toxic *in vivo* (Jain et al, 2010).

The high purity PETIM-(COOH)_16_ dendrimer was then partially glycosylated with d-(+)-glucosamine using EDCI, and complementary analytical techniques used to define its skeletal, generational and substitutional components as previously described (Barata et al, 2011c; Lalwani et al, 2009a, b; Sam et al, 2010; Shaunak et al, 2004). A structural and quantitative NMR analysis showed 4 amide bond-conjugated surface glucosamines per dendrimer (Fig 3D–F and Supporting Information Fig S5). Purity by HPLC-CAD and HPLC-UV (Fig 3G and Supporting Information Figs S6–S8) and capillary electrophoresis (CE; Fig 3H) was 97.2%.

Stability studies showed that PETIM-DG dissolved in water was chemically stable with no loss of bioactivity after storage at 4°C for 9 months, 37°C/100% humidity for 42 days, and heating to 70°C for 1 h (Supporting Information Fig S9). In addition, no *in vivo* toxicity was seen in CD-1 mice that were clinically observed for 24 h after a single oral dosing of PETIM-DG by gastric lavage (*n* = 16) or a single intraperitoneal dosing (*n* = 16) over the dose range 35–175 mg/kg (unpublished observation).

### *In vitro* studies of PETIM-DG with LPS

*In vitro*, PETIM-(COOH)_16_ dendrimer did not block LPS-induced cytokine responses (Supporting Information Fig S9A). Endotoxin-free (<0.06 EU/ml) PETIM-DG at 600 µg/ml was not toxic to cells in whole human blood when cultured at 37°C for 48 h and then analyzed by Coulter flow cytometry for erythrocytes, neutrophils, lymphocytes, monocytes, eosinophils, basophils and platelets (Supporting Information Fig S10). TNF-α release from purified human CD3^+^ T-lymphocytes was <10 pg/ml (EIA limit of assay detection) and did not change when PETIM-DG was added (unpublished observation).

**Figure 4:**
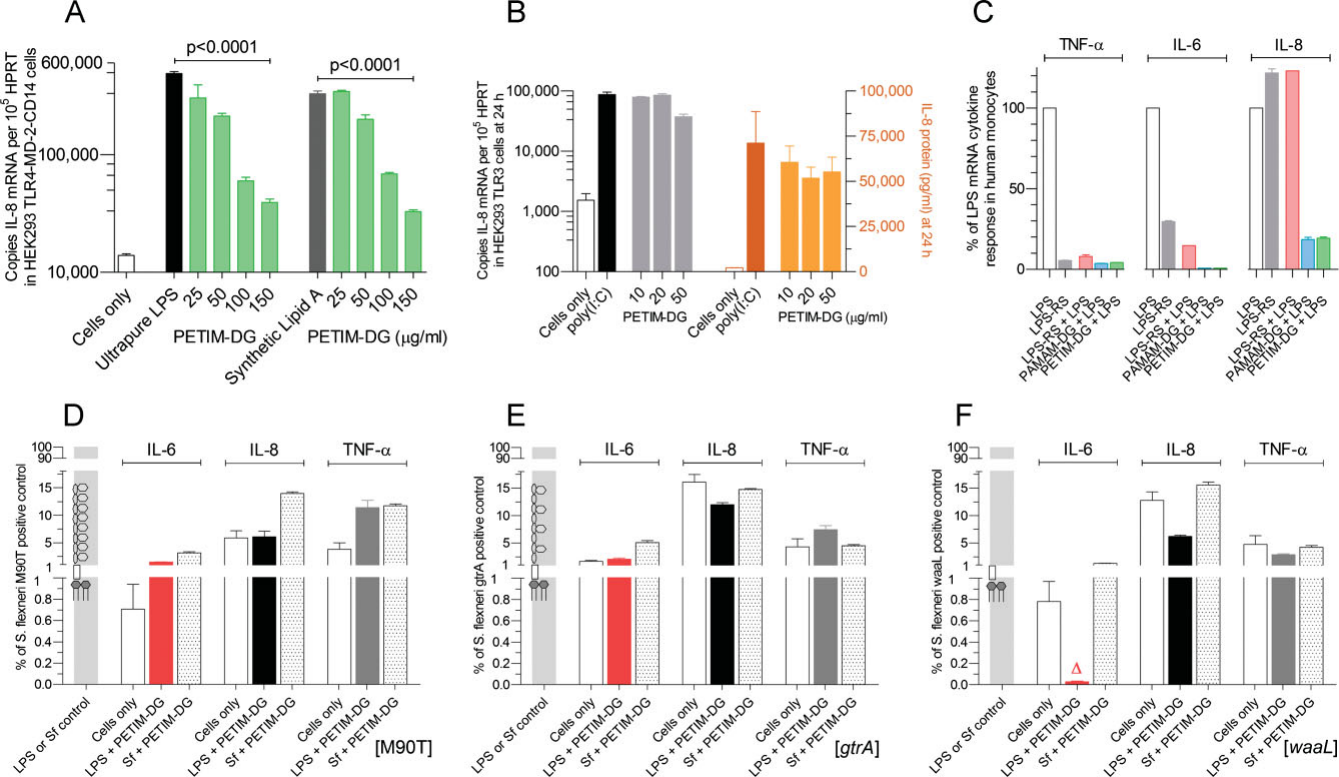
*In vitro* cellular bioactivity of PETIM-DG **A.** PETIM-DG inhibited *S. minnesota* LPS- and synthetic Lipid A-induced IL-8 mRNA expression in HEK293 TLR4-MD-2-CD14-transfected cells with an mRNA ED_50_ = 60 ± 5 µg/ml (4.5 µM). Pooled data (*n* = 6) shown as mean ± sem on a log_10_ scale. *p* Values determined using a two-tailed Mann–Whitney test.**B.** PETIM-DG had no effect on poly(I:C)-induced IL-8 mRNA and protein in HEK293 TLR3-transfected cells at 24 h. Pooled data (*n* = 3) shown as mean ± sem on a log_10_ scale. *p* Values determined using a two-tailed Mann–Whitney test.**C.** Human monocytes were cultured with (i) ultrapure *S. minnesota* LPS (25 ng/ml), (ii) LPS-RS which is the diphosphoryl Lipid A of *R. sphaeroides* (2.5 µg/ml), (iii) LPS-RS (2.5 µg/ml for 1 h) followed by LPS (25 ng/ml), (iv) PAMAM-DG (200 µg/ml for 1 h) followed by LPS (25 ng/ml) and (v) PETIM-DG (200 µg/ml for 1 h) followed by LPS (25 ng/ml). Three hours later, the cells were harvested and RNA extracted. LPS-RS was an antagonist for TNF-α and IL-6 but an agonist for IL-8 mRNA expression. PAMAM-DG and PETIM-DG were antagonists for TNF-α, IL-6 and IL-8. Pooled data (*n* = 3) shown as percentage mean ± sem of the ultrapure *S. minnesota* LPS response which was defined as 100% for each cytokine.**D–F.** Preincubation of human monocytes with PETIM-DG (100 µg/ml) for 1 h inhibited the induction of IL-6, IL-8 and TNF-α in human monocytes by: (i) ultrapure *Shigella* LPS and wild type *S. flexneri* (Sf; M90T strain) (**D**); (ii) ultrapure LPS-*gtrA* (shorter O-chains) and the genetically engineered strain *gtrA* (**E**); (iii) ultrapure LPS-*waaL* (endotoxin component of LPS that binds only to MD-2) and the genetically engineered strain *waaL* (**F**). Δ = 0.029 ± 0.007%. Pooled data (*n* = 3) shown as mean ± sem. *p* < 0.0001; *p* values determined using a two-tailed Mann–Whitney test.

Endotoxin-free (<0.06 EU/ml) PETIM-DG was a dose-dependent antagonist of both ultra-pure *Salmonella minnesota* LPS (Fig 4A—left hand of panel) and of synthetic Lipid A (Fig 4A—right hand of panel) in HEK293 TLR4-MD-2-CD14-transfected cells with an mRNA ED_50_ (PETIM-DG concentration that reduced the *S. minnesota* LPS and synthetic Lipid A positive controls by 50%) of 60 ± 5 µg/ml (4.5 µM). PETIM-DG had no effect on the IL-8 response in HEK293 TLR3-transfected cells using poly(I:C) as the agonist (Fig 4B).

In TLR4-MD-2-CD14-transfected HEK293 cells, ultrapure *S. minnesota* LPS (25 ng/ml) is an MD-2 agonist, and LPS-RS (2500 ng/ml), which is the diphosphoryl Lipid A of *Rhodobacter sphaeroides*, has been reported to be an MD-2 antagonist (Kutuzova et al, 2001; Takayama et al, 1989). We compared the bioactivity of LPS-RS with that of LPS in human monocytes. LPS-RS (used at a 100-fold excess as recommended by the supplier Invivogen) reduced LPS-induced TNF-α expression (92 ± 3% of the LPS-induced control) and IL-6 expression (82 ± 2% of the LPS-induced control; Fig 4C). Surprisingly, in the case of IL-8, LPS-RS acted as an agonist (Fig 4C). This was in contrast to both endotoxin-free PAMAM-DG (200 µg/ml) and PETIM-DG (100 µg/ml), which consistently reduced LPS-induced TNF-α, IL-6 and IL-8 expression (Fig 4C). Taken together, these results showed that PAMAM-DG and PETIM-DG were better antagonists of MD-2 for TNF-α, IL-6 and IL-8 than LPS-RS in primary human monocytes.

When intestinal epithelial Caco-2 cells were used [functionally intact for both TLR4 and intracellular LPS signalling, but without MD-2 (Abreu et al, 2001)], neither LPS nor endotoxin-free PETIM-DG had an effect on cytokine responses (Supporting Information Fig S11). *Shigella* is inefficient at entering gut columnar epithelial cells and does not activate cytosolic sensors and Nod-mediated signalling (Coron et al, 2009). Thus, the absence of enterocyte cell surface MD-2 explained the lack of effect of both LPS and DG. These *in vitro* biological studies showed that DG was an antagonist of MD-2-Lipid A mediated cytokine responses in human monocytes.

### *In vitro* studies of PETIM-DG with bacteria

Polypropyletherimine-DG had no antibacterial activity at 5 mg/ml against *E. coli* (Supporting Information Fig S12). It inhibited the induction of IL-6, IL-8 and TNF-α in human monocytes by: (i) ultrapure *Shigella* LPS and wild-type *S. flexneri* (M90T strain at a multiplicity of infection of 10; Fig 4D); (ii) ultrapure LPS-*gtrA* and the genetically engineered strain *gtrA* (Fig 4E); (iii) ultrapure LPS-*waaL* and the genetically engineered strain *waaL* (Fig 4F). The percentage inhibition of LPS-induced IL-6, IL-8 and TNF-α expression by endotoxin-free PETIM-DG in human monocytes ranged from 85–99.97% and it was greatest for IL-6 at 97.5–99.97%. The mRNA ED_50_ (PETIM-DG concentration that reduced the bacterial positive control by 50%) was 100 ± 7 µg/ml and the protein ED_50_ was 10 ± 1 µg/ml (3.3 µM). Notably, PETIM-DG was 57-fold more potent at suppressing *Shigella waaL* mutant LPS (*i.e.* Lipid A— endotoxin component of LPS that binds only to MD-2)-induced human monocyte IL-6 than PAMAM-DG (Figs 1F and 4F). Taken together, these results suggest that DG is competing with the Lipid A moiety of LPS for MD-2 rather than with the oligosaccharides of LPS, and that, *in vitro*, PETIM-DG is a better antagonist than PAMAM-DG of the MD-2-Lipid A interaction in human monocytes.

### Rabbit model of shigellosis and quantitative RT-PCR transcriptional gene analysis

**Figure 5:**
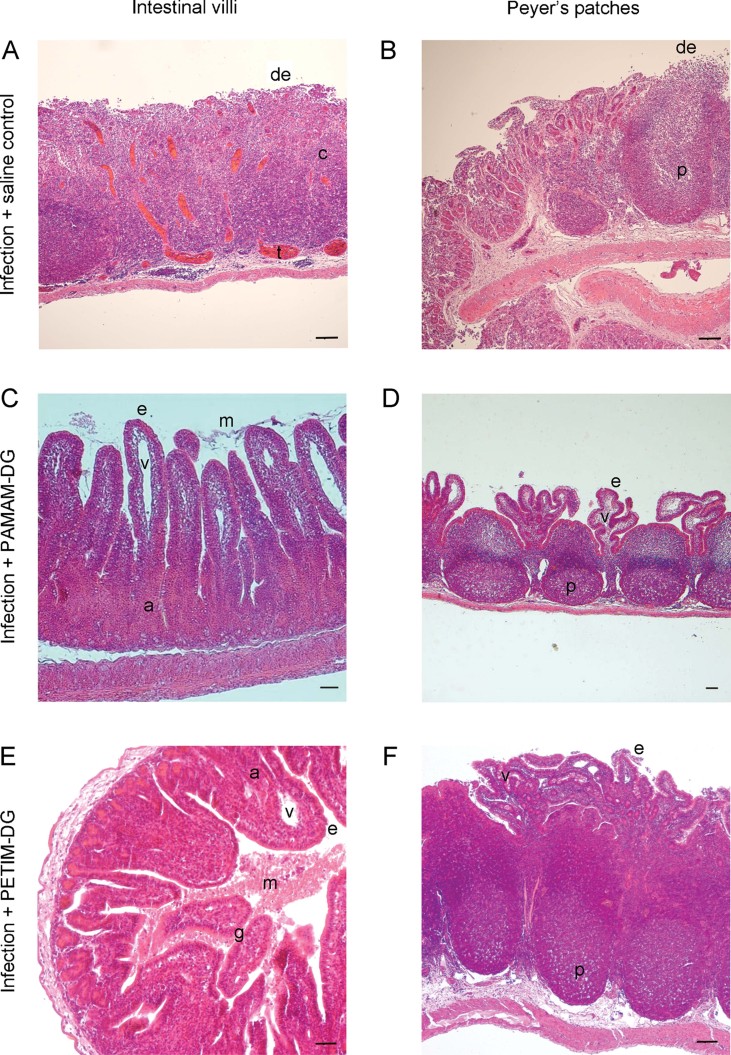
Histology of PAMAM-DG's and PETIM-DG's *in vivo* bioactivity in the rabbit model of shigellosis Sections stained with H&E. Scale bars = 50 µm. Gut intestinal villi after infection with 10^7^ wild type *S. flexneri* and treatment with intraluminal saline control. There was complete loss of surface columnar epithelium and intestinal villous destruction (de) with crypt goblet cell depletion. Sub-mucosal edema and blood vessel thrombosis (t) were associated with a marked neutrophil, lymphocyte and monocyte infiltrate in the gut lamina propria (c). Result from 1 of 4 experiments shown.Peyer's patches after infection with 10^7^ wild type *S. flexneri* and treatment with intraluminal saline control. The Peyer's patches (p) were preserved but their specialized epithelium was lost (de). Result from 1 of 4 experiments shown.Gut intestinal villi after infection with *S. flexneri* and treatment with PAMAM-DG. Mucus (m) was present, and the columnar epithelium (e) and intestinal villi (v) were both intact. This protected the lamina propria (a) from any damage. Result from 1 of 6 experiments shown.Peyer's patches after infection with *S. flexneri* and treatment with PAMAM-DG. The columnar epithelium (e) and intestinal villi (v) were both intact. This protected the Peyer's patches (p) from any damage. Result from 1 of 6 experiments shown.Gut intestinal villi after infection with *S. flexneri* and treatment with PETIM-DG. Mucus (m) was present, and the columnar epithelium (e) and intestinal villi (v) were both intact. This protected the lamina propria (a) from any damage. Result from 1 of 6 experiments shown.Peyer's patches after infection with *S. flexneri* and treatment with PETIM-DG. The columnar epithelium (e) and intestinal villi (v) were both intact. This protected the Peyer's patches (p) from any damage. Result from 1 of 6 experiments shown.

As the human MD-2 amino acid residues binding LPS and the proposed binding sites for PAMAM-DG (Barata et al, 2011b) in small animals are most conserved in rabbit (Human/rabbit MD-2: K91K;R96K;S98S;Y102Y;R106R;K109K;T112T;N114N;T116T;S118P), a rabbit intestinal loop model of shigellosis was used to determine the bioactivity of PAMAM-DG (Perdomo et al, 1994; Raqib et al, 1995; Singer & Sansonetti, 2004). Rabbits are phylogenetically, anatomically and physiologically more closely related to primates than rodents, and, in many cases, lead to better modelling of human disease states. In the context of shigellosis, the well-defined histopathology of this animal model (Fig 5A and B) mirrors the clinical observation that the IL-6- and IL-8-mediated inflammatory response in shigellosis is localized to the gut (Coron et al, 2009; Islam et al, 1994; Schnupf & Sansonetti, 2012; Sperandio et al, 2008).

However, the limited availability of enzyme immuno-assay reagents for rabbit cytokines and chemokines and for the immuno-histochemistry of both cells and tissue sections means that conventional histological examination remains the gold standard for analyzing the rabbit's immune response. Also rabbit microarrays are not available. It is in this context that we have used quantitative polymerase chain reaction (after reverse transcription) (RT-PCR) as a sensitive and rapid means by which to monitor the spectrum of the host immune response. While quantitative PCR lacks the large-scale throughput of microarrays, it is considered to be the gold standard for gene expression analysis because of its ease of use, high detection sensitivity and wide linear dynamic range. The rabbit ileal loop model of shigellosis is accurate both morphologically and with respect to transcriptional gene activation during the course of an infection (Schnupf & Sansonetti, 2012).

For our RT-PCR assays, we designed and created human and rabbit multi-gene quantification plasmids as standards and used SYBR Green hot-start PCR-based amplification rather than a fluorescent probe-based detection system because the cost differential for multiple gene analysis in many samples can be considerable. Hypoxanthine phosphoribosyltransferase (HPRT) was chosen as the reference ‘housekeeping’ gene because it is a reliable medium-to-low abundance mRNA transcript as previously described (Corware et al, 2011; Shaunak et al, 2004). The principle advantage of using a multi-gene plasmid-based quantification standard rather than a comparative ΔCt or a Δ-ΔCt method was that our approach did not depend upon equal amplification efficiencies for both the target and the ‘housekeeping’ HPRT reference gene. In addition, the assay reliably and reproducibly generates real mRNA copy numbers that can be directly correlated with the amount of starting RNA or cell number. This enables a considerably more accurate cross comparison of the mRNA levels of different chemokines and cytokines across multiple experiments.

### Bioactivity of a large PAMAM-DG molecule in shigellosis

The *in vivo* studies started with the intraperitoneal administration of 10 mg/ml of endotoxin-free (<0.06 EU/ml) PAMAM-DG (20 mg/kg) dissolved in isotonic saline to rabbits during the surgical procedure to create and infect the ligated ileal loops with *S. flexneri*. This initial experiment showed that 20 mg/kg of intraperitoneal PAMAM-DG was bioactive in reducing gut wall tissue injury and in reducing IL-6 expression in Peyer's patches (Supporting Information Fig S13). The intraperitoneal route of administration was not studied further because one of our key aims was to make an orally bioavailable and bioactive molecule. Studies in rats have previously shown that 96% of an orally administered tritiated dendrimer with 16 peripheral groups and a diameter of 2.5 nm was retained in the gut (Florence et al, 2000). The peak level of tritiated dendrimer in the small and large bowel was seen at 6 h with <1% remaining in the gut at 24 h (Sakthivel et al, 1999). These observations led us to conclude that in our rabbit model of shigellosis, administration of DG into the ligated ileal loops would mean that most of it would remain in the gut lumen.

Wild-type *S. flexneri* (M90T strain, 10^7^ bacteria/loop) and endotoxin-free PAMAM-DG were administered into ligated ileal loops with Peyer's patches; *i.e.* aggregated lymphoid follicles that are separated from the gut lumen by a specialized follicle-associated epithelium. In the mouse Peyer's patch, 60% of the cells are B-cells (B220^+^), 25% are T-cells (CD3^+^), 10% are dendritric cells (CD11c^+^), 4.5% are macrophages (F4/80^+^) and neutrophils (Ly-6G^+^), and 0.5% are CD3^+^CD4^+^FoxP3^+^ T regulatory (T_reg_) cells (Barreau et al, 2007). Typically, four ligated loops with Peyer's patches were created in each rabbit. Our studies were focused on Peyer's patches because of the intimate association of their overlying epithelium with pathogenic bacteria in the earliest stages of an infectious diarrhoeal disease (Inman and Cantey, 1984; Takeuchi, 1967; Takeuchi et al, 1968, 1978). The animal infection experiments with PAMAM-DG were undertaken for a 12 h period.

**Figure 6:**
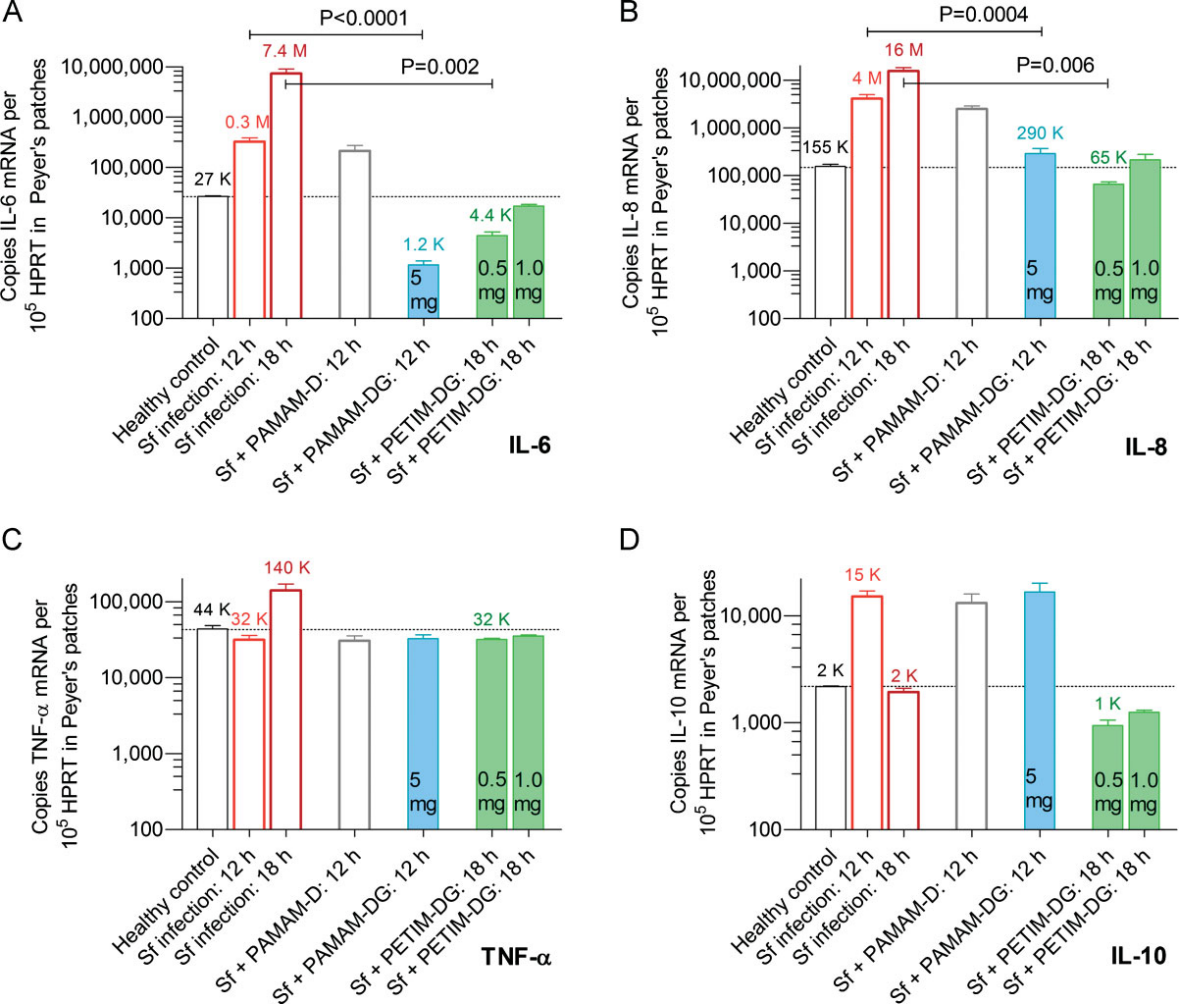
PAMAM-DG and PETIM-DG reduced IL-6 and IL-8 expression but not TNF-α or IL-10 expression in Peyer's patches upon *S. flexneri* infection IL-6 mRNA levels increased during the course of a wild type *S. flexneri* (Sf) infection. This cytokine increase was reduced by 5 mg/loop of PAMAM-DG at 12 h and by 0.5 mg/loop of PETIM-DG at 18 h. PAMAM-dendrimer (PAMAM-D) had no effect.IL-8 mRNA levels increased during the course of a wild type *S. flexneri* (Sf) infection. This cytokine increase was reduced by 5 mg/loop of PAMAM-DG at 12 h and by 0.5 mg/loop of PETIM-DG at 18 h. PAMAM-dendrimer (PAMAM-D) had no effect.TNF-α mRNA levels increased 3.2-fold at 18 h during the course of a wild-type *S. flexneri* infection. PAMAM-dendrimer, PAMAM-DG and PETIM-DG had no effect on TNF-α mRNA levels compared to healthy control rabbits.The infection-associated increase in IL-10 mRNA levels at 12 h was not reduced by PAMAM-DG. At 18 h, IL-10 mRNA had fallen to the healthy control level in all groups. Pooled data (*n* = 4) shown as mean ± sem on a log_10_ scale. K = thousand, M = million. *p* Values determined using a two-tailed Mann–Whitney test.

In rabbits receiving 5 mg/loop of endotoxin-free (<0.06 EU/ml) PAMAM-DG, there was minimal damage to surface epithelium and intestinal villi at 12 h, and crypt goblet cells were preserved (Fig 5C and D). This PAMAM-DG-mediated protection of columnar epithelium was associated with a 282-fold reduction in IL-6 (Fig 6A) and a 14-fold reduction in IL-8 expression in Peyer's patches (Fig 6B). At 12 h, TNF-α expression did not change when compared to both healthy rabbits and infected untreated rabbits (Fig 6C). IL-10 (anti-inflammatory cytokine) did not change when compared to infected untreated rabbits at 12 h (Fig 6D). Interferon-β (TRIF-dependent mediator of dendritic cell maturation) expression did not change significantly in infected treated rabbits (5430 ± 760 mRNA/10^5^ HPRT) compared to infected untreated rabbits (3700 ± 810 mRNA/10^5^ HPRT; *n* = 3; *p* = 0.06). β-defensin (an epithelial cell anti-microbial peptide) expression also did not change in infected treated rabbits (3570 ± 100 mRNA/10^5^ HPRT) compared to infected untreated rabbits (3940 ± 1040 mRNA/10^5^ HPRT; *n* = 3; *p* = 0.7). CD3^+^ mRNA expression increased threefold in infected and PAMAM-DG-treated rabbits (1.53 ± 0.11 × 10^6^ copies of CD3 mRNA/10^5^ HPRT) over infected untreated control rabbits [0.50 ± 0.03 × 10^6^ copies of CD3 mRNA/10^5^ HPRT (*n* = 3; *p* = 0.01)].

No clinical toxicity, or macroscopic or microscopic organ based tissue toxicity was seen in the rabbits (unpublished observation). Although intestinal bacterial counts increased from 10^7^ to 5 × 10^8^ colony forming units because PAMAM-DG has no anti-bacterial activity (Supporting Information Figs S2 and S14), Peyer's patch *Shigella* IcsA expression (an indicator of bacterial gut wall invasion) fell by 82 ± 8% in PAMAM-DG-treated rabbits (5 mg/loop) compared to infected untreated animals (Supporting Information Fig S15). Gut tissue immunohistochemistry for *Shigella* LPS confirmed this large reduction in bacterial gut wall invasion in rabbits treated with PAMAM-DG (Supporting Information Fig S16).

### Bioactivity of a small PETIM-DG molecule in shigellosis

The time period of animal infection with *S. flexneri* was extended to 18 h in the PETIM-DG studies. These *in vivo* rabbit studies showed that endotoxin-free (<0.06 EU/ml) PETIM-DG was bioactive against *S. flexneri* at 0.5 mg/loop (600 µg/kg) with complete protection of gut surface columnar epithelial cells and intestinal villi. Crypt goblet cell preservation suggested that there was no loss of mucins (Fig 5E and F). In addition, there was a 1653-fold reduction in IL-6 (Fig 6A) and a 238-fold reduction in IL-8 expression (Fig 6B) in Peyer's patches compared to the untreated control. At 18 h, TNF-α expression did not change when compared to healthy rabbits and was lower than in infected untreated rabbits (Fig 6C). IL-10 expression did not change when compared to both healthy rabbits and infected untreated rabbits at 18 h (Fig 6C). Interferon-β expression did not change significantly in infected treated rabbits (7400 ± 3560 mRNA/10^5^ HPRT) compared to healthy control rabbits (3570 ± 860 mRNA/10^5^ HPRT; *n* = 3; *p* = 0.4), and β-defensin expression also did not change significantly in infected treated rabbits (18,000 ± 1100 mRNA/10^5^ HPRT) compared to healthy control rabbits (12,400 ± 860 mRNA/10^5^ HPRT; *n* = 3; *p* = 0.3). The result for β-defensin is notable because the antibacterial peptides LL-37 and human β-defensin-1 are reduced in human shigellosis and it can take 60 days for them to be restored in surface epithelial cells (Islam et al, 2001). Taken together, these results suggest that PETIM-DG was still bioactive 18 h after the onset of infection at a 2.5-fold (molar basis — 0.15 µM/loop) to 10-fold (weight basis — 0.5 mg/loop) lower concentration than PAMAM-DG (molar basis — 0.38 µM/loop; weight basis — 5 mg/loop). No clinical, or macroscopic or microscopic organ based tissue toxicity was seen in PETIM-DG treated rabbits (unpublished observation).

**Figure 7:**
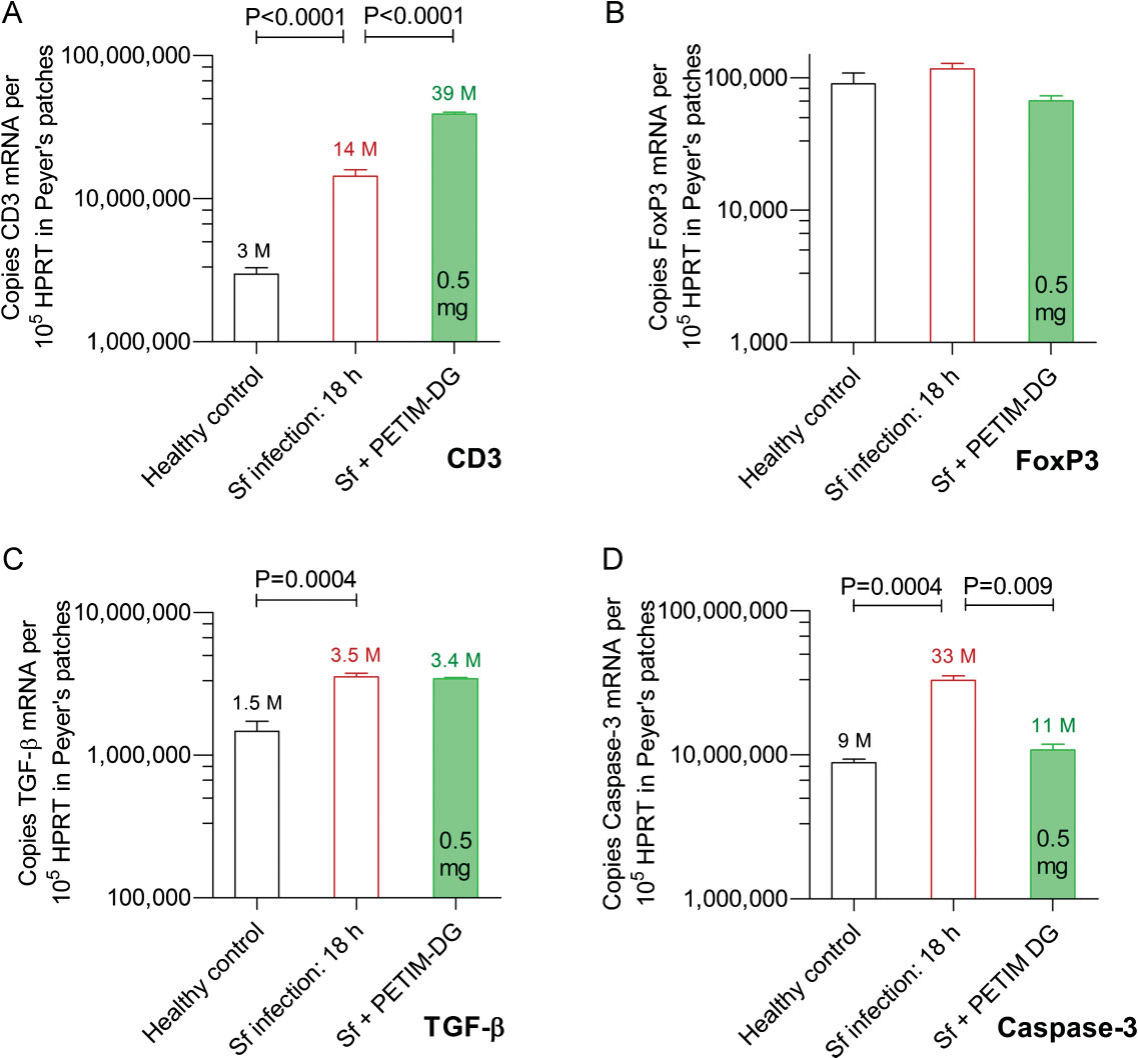
CD3, Fox P3, TGF-β and caspase-3 expression in Peyer's patches CD3^+^ mRNA levels increased upon *S. flexneri* (Sf) infection at 18 h post-infection, and increased further upon infection and PETIM-DG treatment.There was no change in FoxP3^+^ mRNA levels (an indicator of T_reg_ cells) upon infection or with PETIM-DG treatment.The 2.3-fold infection-associated increase in TGF-β mRNA levels was not affected by PETIM-DG.Caspase-3 mRNA levels (an indicator of apoptosis) increased 3.7-fold with wild type *S. flexneri* (Sf) infection at 18 h. This increase was prevented by 0.5 mg/loop of PETIM-DG. Pooled data (*n* = 3) shown as mean ± sem on a log_10_ scale. M = million. *p* Values determined using a two-tailed Mann–Whitney test.

CD3^+^ mRNA expression increased significantly in infected and PETIM-DG-treated rabbits over uninfected control rabbits (13-fold), and slightly over infected untreated control rabbits (2.7-fold; Fig 7A). Forkhead box P3 (FoxP3) expression was not altered by either *S. flexneri* infection or by PETIM-DG treatment (Fig 7B) while transforming growth factor-β (TGF-β) expression increased 2.3-fold upon infection and was not altered by PETIM-DG treatment (Fig 7C). Infiltration of the surface epithelium and lamina propria by γδ^+^ CD4^+^ and CD8^+^ T-cells occurs in human shigellosis (Islam & Christensson, 2000; Raqib et al, 1994) with CD3^+^CD4^+^FoxP3^+^ T regulatory (T_reg_) cells maintaining gut tolerance by suppressing local antigen specific immune responses to commensal bacteria (Sellge et al, 2010). In the steady state, T_reg_ cells are dependent upon local dendritic cell- and TGF-β-induced signals (Izcue et al, 2009), but when proinflammatory cytokines increase, they switch to become dependent upon monocyte/macrophage-derived IL-10 (Murai et al, 2009; Unutmaz & Pulendran, 2009). Our results suggest that DG alters the recently recognized balance between cytokine-mediated tissue injury and T cell-mediated tolerance in the gut (Ismail et al, 2009, 2011) so that epithelial gut wall integrity is preserved and bacterial gut wall invasion is minimized. This conclusion is also supported by the observation that shigellosis-induced apoptosis of gut lamina propria CD3^+^ T-cells, CD15^+^ granulocytes and CD56^+^ macrophages (Lembo-Fazio et al, 2011; Raqib et al, 2002; Zychlinsky et al, 1996) was prevented by PETIM-DG as measured by caspase-3 mRNA expression (Fig 7D).

**Figure 8:**
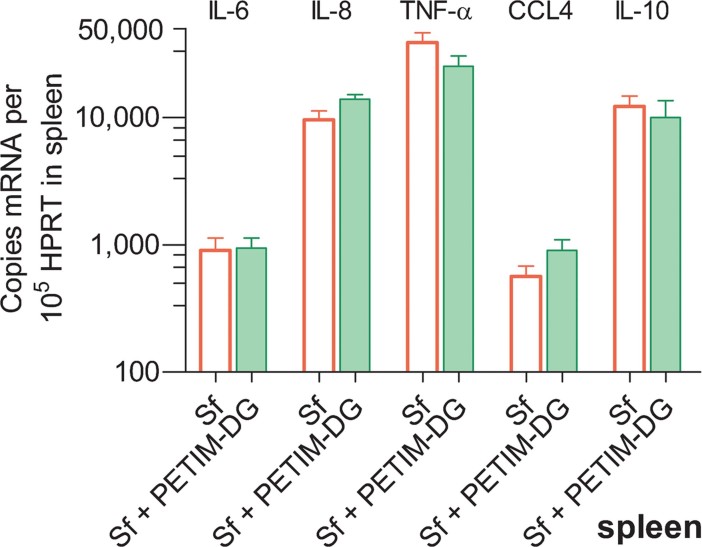
Spleen cytokines IL-6, IL-8, TNF-α, CCL4 and IL-10 mRNA expression was lower in spleen than in Peyer's patches at 18 h. The levels were not affected by local administration of PETIM-DG into the ileal loops. *S. flexneri* (Sf). Pooled data (*n* = 3) shown as mean ± sem on a log_10_ scale.

Finally, there was no change in IL-6, IL-8, TNF-α, CCL4 or IL-10 expression in the spleen (Fig 8). This confirmed the gut localization of the host cytokine response to shigellosis.

## DISCUSSION

**Figure 9:**
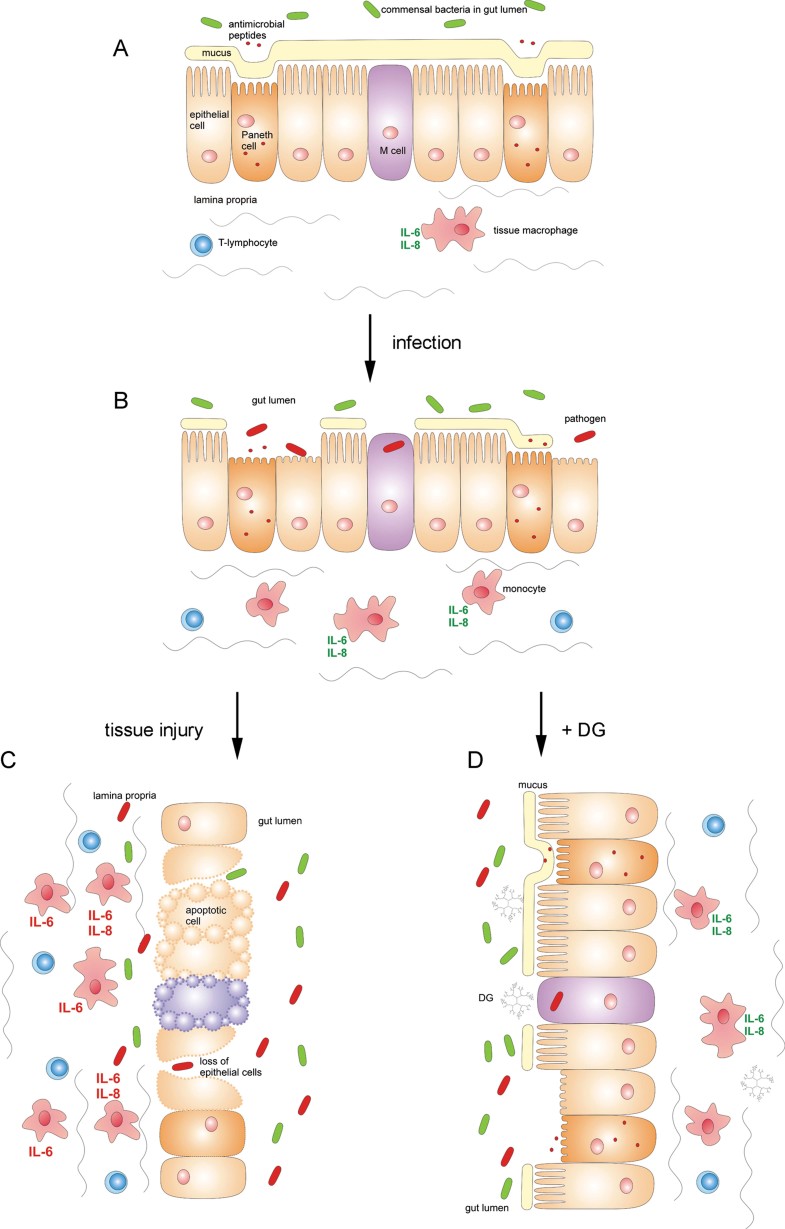
Illustration of the proposed mechanism for epithelial gut wall damage in shigellosis and the protective effect of DG In the normal and healthy state, local cytokine production in response to commensal gut bacteria protects epithelial cells and macrophages from apoptosis.The initial host response to *S. flexneri* infection is increased local IL-6 and IL-8 with minimal bystander damage to epithelial gut wall integrity. This is a physiologically appropriate response.Severe infection leads to an excessive pathogen-triggered IL-6 and IL-8 gut cytokine storm that destroys the epithelial gut wall's integrity. Bacterial invasion follows with apoptosis of epithelial cells, neutrophils, lymphocytes, monocytes and tissue macrophages.Treatment with DG alters the balance between cytokine mediated tissue injury and T-cell mediated tolerance so that epithelial gut wall integrity is preserved and bacterial gut wall invasion is minimized.

Our integration of modelling studies with biological experiments led to a 75% reduction in the molecular weight of the DG from 13.6 to 3.3 kDa and an increase in its purity to 97%. This synthetic baby-bio (SBB; Barata et al, 2011b, c) prevented the pathogen-induced and cytokine-mediated injury to gut columnar epithelium and intestinal villi that is associated with increased IL-6 and IL-8 expression in shigellosis. Complete protection of the epithelial gut wall in rabbits by PETIM-DG at a dose 0.5 mg/loop (600 µg/kg) prevented crypt goblet cell depletion and submucosal edema, and also minimized bacterial gut wall invasion. An illustration of the proposed mechanism for epithelial gut wall damage in shigellosis and the protective effect of DG is shown in Fig 9. In addition, PETIM-DG did not interfere with endogenous anti-microbial peptides, TRIF-dependent dendritic cell maturation or CD3 and FoxP3 expression. The shigellosis-induced increase in caspase-3 was also prevented. This suggests that the key functions of IL-10 and TGF-β in controlling TLR-mediated activation of antigen presenting cells in the gut were not disrupted by PETIM-DG.

These animal model-based results are consistent with two important clinical observations for intestinal diarrhoeal diseases. The first is that severe shigellosis (*i.e.* hemolytic uremic syndrome, thrombocytopenia and severe colitis >1 week) is associated with elevated IL-6 rather than TNF-α (Harendra de Silva et al, 1993). The second is that only acute invasive bacterial enteritis in humans, such as that due to *Shigella* spp., enterohemorrhagic *E. coli*, *Salmonella* spp. and *Campylobacter* spp. have been shown to be associated with increased IL-6 and, to a lesser degree, IL-8 expression (Vaisman et al, 2003). In this context, it is interesting to note that gut pathogens are often found in very close proximity to the gut surface epithelium, a position from which they can stimulate local pro-inflammatory cytokine responses. Electron microscopy studies of the earliest stages of *S. flexneri*, enterohemorrhagic *E. coli* and *Salmonella typhimurium* infections have demonstrated the intimate nature of this association between pathogenic bacteria and the specialized epithelium overlying Peyer's patches (Inman and Cantey, 1984; Takeuchi, 1967; Takeuchi et al, 1968, 1978).

Our molecular modelling studies suggested that PETIM-DG's antagonist activity depended upon the formation of co-operative electrostatic interactions with two key residues that line the entrance to MD-2's hydrophobic pocket; the first is Ser^118^ to which the 4′-phosphate on the diglucosamine of Lipid A binds, and the second is Tyr^102^ to which TLR4 binds. The binding of PETIM-DG to MD-2 blocks the entry of the acyl chains of the Lipid A of LPS into MD-2's hydrophobic pocket. This prevents the formation of the primary MD-2-LPS complex and the secondary TLR4-MD-2-LPS receptor complex. The β cup topology of the lipid binding pocket of MD-2 reflects its need to accommodate large and structurally diverse ligands. Such promiscuity is an important evolutionary feature and reflects the need for MD-2 to be able to recognize all of the diverse LPS structures synthesized by individual species of Gram-negative bacteria, and also the binding of those endogenous host derived ligands that act as alert signals for tissue injury including enzyme derived fragments of hyaluronan from injured extracellular matrix (Atala et al, 2010; O'Neill, 2005). In this context, we believe that our studies have defined the first non-lipid based synthetic antagonist of MD-2 using both molecular modelling studies and animal model based studies of cytokine responses. Using a similar approach, it should now be possible to computationally design additional dendrimer based antagonists for other Toll-like receptors. This could provide new opportunities for treating a range of infectious, inflammatory and malignant diseases in whose pathogenesis Toll-like receptors play an important role.

If our results are reproduced in non-human primates (the only other natural host for shigellosis), small, well defined, cost-effective and orally delivered glycosylated dendrimers could become a useful new therapeutic addition for a spectrum of other cytokine-mediated inflammatory diarrhoeas including infection with enterohemorrhagic *E. coli* (Buchholz et al, 2011), *Salmonella* sp. (Arpia et al, 2011), *Campylobacter* sp. (Rathinam et al, 2009) and potentially even *Clostridium difficile*, which has recently been shown to also engage TLR4 (Ryan et al, 2011). Furthermore, this non-antibiotic-based approach to treating infectious diarrhoeas is one to which pathogen resistance will likely not arise. It also supports the growing recognition from both human and animal studies that it is the pathogen-triggered and IL-6-mediated gut cytokine storm that destroys epithelial gut wall integrity, promotes bacterial tissue invasion, and leads to severity of clinical disease seen. Our results suggest that small and orally delivered glycosylated dendrimers could be therapeutically useful for preventing gut wall tissue damage in a wide spectrum of infectious diarrhoeal diseases.

**The paper explained**

PROBLEM:

Intestinal pathogens cause life threatening diarrhoeas in hundreds of millions of people worldwide. They use the host's gut cytokine storm and the associated tissue injury to promote their tissue invasion. IL-6 and IL-8 are primarily responsible for the severity of epithelial gut wall damage seen in both humans and animals. This tissue injury is not prevented by antibiotic treatment. We therefore sought a new molecule and approach by which to prevent this acute cytokine-mediated damage to gut epithelium.

RESULTS:

The TLR4-MD-2-LPS complex is involved in mediating this pathogen-induced tissue damage, and shigellosis is a well-defined disease model for its study. Chemically defined and high purity DG-based molecules were synthesized and tested *in vitro* and *in vivo*. A large (13.6 kDa) and small (3.3 kDa) DG molecule prevented both epithelial gut wall damage and intestinal villous destruction when given locally into the gut. This protection was associated with reduced IL-6 and IL-8 in the Peyer's patches; *i.e.* gut associated lymphoid tissues. Taken together, this suggests that treatment with DG alters the balance between cytokine mediated tissue injury and T-cell mediated tolerance to preserve epithelial gut wall integrity and minimize bacterial gut wall invasion.

IMPACT:

With rapidly increasing bacterial virulence and antibiotic resistance worldwide, small, well defined, cost-effective and orally delivered glycosylated dendrimers could become a useful new therapeutic addition for a wide spectrum of cytokine mediated inflammatory diarrhoeas. They include infections with *Shigella* spp., enterohemorrhagic *E. coli*, *Salmonella* sp., *Campylobacter* sp., and potentially *C. difficile*. The non-antibiotic based approach described here for treating infectious diarrhoeas supports the recent and growing recognition that the pathogen-triggered and local IL-6 cytokine storm destroys epithelial gut wall integrity, promotes bacterial gut wall invasion, and leads to severity of clinical disease seen. Small and orally delivered DG could be therapeutically useful for preventing this tissue damage in a wide spectrum of infectious diarrhoeal diseases.

## MATERIALS AND METHODS

### Molecular modelling, chemical synthesis and analytical studies

Longer molecular dynamics with explicit solvent and molecular docking were performed as previously described (Barata et al, 2011a, b, c). The anionic G3.5 PAMAM dendrimer (diaminobutane core) was synthesized by Dendritic Nanotechnologies Michigan. PAMAM-(COONa)_64_ dendrimer: ^1^H NMR (500 MHz, D_2_O, 303K): *δ* 3.55 − 3.50 (br m, 65H), 3.38 − 3.34 (br m, 56H), 3.18 − 3.10 (br m, 120H), 3.05 − 3.00 (br m, 62H), 2.91 − 2.86 (br m, 123H), 2.72 − 2.67 (br m, 64H), 2.57 − 2.47 (br m, 244H), 1.56 (br s, 4H). ^13^C NMR (125 MHz, D_2_O, 303K): *δ* 179.5, 175.2, 174.6, 51.3, 50.3, 48.9, 48.8, 36.7, 35.6, 32.5.

The anionic G3 PETIM dendrimer was synthesized as previously described with minor modifications (Jain et al, 2010; Jayamurugan & Jayaraman, 2006; Krishna and Jayaraman, 2003). PETIM-(COOH)_16_·14HCl dendrimer: ^1^H NMR (500 MHz, D_2_O, 303K): *δ* 3.71 − 3.64 (m, 52H), 3.59 − 3.55 (m, 32H), 3.43 − 3.39 (m, 16H), 3.35 − 3.30 (m, 36H), 3.03 − 2.99 (m, 32H), 2.16 − 2.04 (m, 52H). ^13^C NMR (125 MHz, D_2_O, 303 K): *δ* 174.1, 68.0, 67.7, 67.5, 52.3, 50.8, 50.7, 49.5, 28.4, 23.6, 23.5, 23.3.

d-(+)-glucosamine (purity 99%) was conjugated to both the PAMAM dendrimer's carboxylic acid groups and the PETIM dendrimer's carboxylic acid groups using EDCI as previously described (Sam et al, 2010; Shaunak et al, 2004). PAMAM-DG: ^1^H NMR (500 MHz, D_2_O, 303K): *δ* 5.25 (d, *J* = 3.5 Hz, 5H), 3.96 − 2.99 (m, 555H), 2.93 − 2.61 (m, 235H), 2.11 − 2.02 (m, 10H), 1.84 (br s, 4H), 1.24 − 1.19 (m, 15H).

PETIM-DG: ^1^H NMR (500 MHz, D_2_O, 303K): *δ* 5.26 − 5.23 (m, 3H), 3.96 − 3.04 (m, 218H), 2.93 − 2.88 (m, 30H), 2.69 − 2.66 (m, 14H), 2.18 − 1.90 (m, 64H), 1.27 − 1.19 (m, 8H), 1.11 (t, *J* = 7.4 Hz, 2H). ^13^C NMR (125 MHz, D_2_O, 303 K): *δ* 177.9, 177.7, 177.5, 174.2, 173.4, 172.5, 172.2, 172.0, 94.8, 90.8, 76.0, 73.9, 73.8, 71.7, 70.8, 70.2, 70.0, 67.7, 67.6, 67.4, 60.8, 60.7, 57.0, 56.7, 55.4, 55.0, 54.2, 54.1, 50.9, 50.8, 50.6, 49.7, 49.5, 49.4, 49.3, 42.9, 42.8, 41.7, 40.2, 37.3, 36.5, 35.9, 35.0, 30.7, 30.2, 30.1, 30.0, 29.5, 29.2, 25.0, 24.3, 23.6, 14.5, 13.6, 13.4.

The NMR-based analytical methods used for compound identity included 1D ^1^H NMR in 100% D_2_O and 90:10 H_2_O:D_2_O, 2D ^1^H–^13^C HSQC NMR and 2D ^1^H–^1^H ROESY NMR spectra (500/600/800 MHz Bruker). The MS based analytical methods used for compound identity included MALDI-TOF-MS, trimethylsilyl gas chromatography–mass spectrometry (TMS GC–MS), and esterification (methanolic-hydrochloric acid) of the peripheral carboxylic acids. HPLC with CAD and UV detection at 214 nm, as well as CE (pH 8.55–9.51) were used to determine compound purity as previously described (Barata et al, 2011c; Lalwani et al, 2009a, b; Shaunak et al, 2004).

### *In vitro* biology

Human monocytes (10^6^/ml) were prepared and used as previously described (Corware et al, 2011; Sabroe et al, 2002; Shaunak et al, 2004) with: (i) ultrapure *S. minnesota* LPS (25 ng/ml, Invivogen)—which is an MD-2 agonist; (ii) ultrapure *Shigella* LPS and Lipid A (25 ng/ml)—which are both MD-2 agonists; (iii) synthetic Lipid A (100 ng/ml, Peptides International)—which is an MD-2 agonist and (iv) LPS-RS (2.5 µg/ml, Invivogen {*i.e.* 100-fold of the LPS concentration used}) which is the diphosphoryl Lipid A from *R. sphaeroides*, and which has been reported to be an MD-2 antagonist in TLR4-transfected cells (Kutuzova et al, 2001).

Genetically engineered strains of the *S. flexneri* organisms *gtrA* and *waaL* were constructed and cultured as previously described (West et al, 2005), and ultrapure LPS was also prepared from each strain. The bacteria were cultured with human monocytes at a multiplicity of infection of 10 for 1 h followed by gentamicin for 2 h. Anti-TLR4 antibody (HTA125) and protein EIAs were from eBioscience.

### *In vivo* biology

Animal studies were performed under a French Ministry of Agriculture licence for animal experimentation no 75-305. They took account of the 3Rs of replacement, reduction and refinement, and were performed in accordance with EU laws and regulations.

Ligated ileal loop segments of 5 cm with a Peyer's patch were surgically created in 1 h in New Zealand White rabbits (*Oryctolagus cuniculus*) weighing 2.4–2.7 kg as previously described (Perdomo et al, 1994; Schnupf & Sansonetti, 2012). Typically, four ligated loops with Peyer's patches could be created in each rabbit. Wild-type *S. flexneri* (M90T) in exponential phase growth (10^7^ bacteria) were used to infect each loop, endotoxin free (<0.06 EU/ml) DG added, and the abdomen closed. The DG dose range was 0.1–50 mg/loop in 1 ml normal saline. Rabbits were culled at 12 h for the PAMAM-DG experiments and at 18 h for the PETIM-DG experiments. Tissues were harvested for histology [haematoxylin–eosin (H&E) and immuno-histochemistry], and RNA using TriReagent. Loop fluid bacterial titres were determined using Congo red plates.

The specialized epithelial cells overlying the lymphoid follicles called Peyer's patches are the first to be damaged in an infectious diarrhoea. Mild gut wall damage was histologically defined as an intact epithelial lining, minimal goblet cell depletion and a slight increase in the number of neutrophils and lymphocytes in the surface epithelium and lamina propria. Severe gut wall damage was histologically defined as a destroyed surface epithelium, severe goblet cell depletion and a marked increase in the number of neutrophils and lymphocytes in the crypt epithelium and lamina propria (Islam et al, 1994; Islam & Christensson, 2000; Islam et al, 2001; Raqib et al, 1994; Schnupf & Sansonetti, 2012).

#### Design and construction of human and rabbit multi-gene quantification plasmids for RT-PCR

messenger RNA quantification was performed by real-time PCR using custom made human and rabbit quantification multi-gene plasmid standards based upon methods and primer pairs as previously described (Corware et al, 2011; Schnupf & Sansonetti, 2012; Shaunak et al, 2004). Using this approach to quantitative RT-PCR, it was possible to reliably and reproducibly identify twofold changes in absolute mRNA copy number.

For the human multi-gene quantification plasmid, cDNA was made from human monocytes treated with LPS (25 ng/ml) for 3 h. This induced expression of otherwise low copy number chemokine and cytokine mRNA transcripts. The cDNA was amplified by PCR using the human primer pairs shown in Supporting Information Table S1. A single human multi-gene plasmid was made by sequential ligation and cloning of the authenticated (by sequencing) PCR amplified products of β-actin, HPRT, interferon-1β, interferon-γ, IL-2, IL-6, IL-8, MIF, MIP-2α, MIP-1α (CCL3), MIP-1β (CCL4), chemokine (C-C motif) ligand 5 (CCL5) and TNF-α into a pGEM-TA (Promega) vector. The plasmid was sequenced to confirm its identity and linearized with *Sac*II.

For the rabbit multi-gene quantification plasmids, cDNA was made from the Peyer's patches of Shigella infected rabbits. This induced expression of otherwise low copy number chemokine and cytokine mRNA transcripts. The cDNA was amplified by PCR using the rabbit primer pairs shown in Supporting Information Table S1. Two multi-gene plasmid standards were constructed by ligation of the amplified and authenticated (by sequencing) PCR products as described above. The first plasmid contained GAPDH, IL-6, IL-8, MIP-1β (CCL4) and TNF-α cloned into a TOPO4 (Invitrogen) vector that was linearized with *Not*I. The second multi-gene plasmid contained HPRT, interferon-1β, interferon-γ, IL-12 p35, IL-12 p40 and IL-10 cloned into a pGEM-TA vector and linearized with *Not*I. All other genes studied were cloned as individual plasmids into cloning vectors pGEM TA, TOPO4 or pDrive (Qiagen) and the amplified target gene number normalized using the ‘housekeeping’ HPRT reference gene.

#### Sample extraction and quantitative RT-PCR for mRNA expression

All samples were lysed with Tri-Reagent (Sigma) for RNA extraction. For the rabbit tissue based samples, 1 ml aliquots of Tri-Reagent lysates were centrifuged in 2 ml Max Tract tubes (Qiagen) at 12,000*g* for 10 min, the aqueous phase above the wax barrier removed, and the RNA precipitated as per the manufacturer's instructions. For tissue culture derived samples, the Max Tract tube step was omitted. Eight hundred ng of purified RNA was then reverse transcribed with a QuantiTect RT kit (Qiagen) which incorporated a DNAse digestion step. The cDNAs were then diluted 1:4 with RNAse free water.

Standard curves were generated by serial 10-fold dilutions of 10 to 10^7^ copies of the relevant plasmid in water containing 5 ng/µl Lambda DNA. Triplicate 2 µl aliquots of sample cDNA and 2 µl aliquots of each of the standard curve dilutions were amplified with each gene specific primer pair in Jumpstart Sybr Green mix (Sigma) in the same PCR run in a Corbett Research Rotor-Gene 3000 machine. The fluorescence acquisition temperature was set at 5–7°C below the melting temperature of each amplified product in order to eliminate any signal from primer-dimers. The authenticity of each amplified product was confirmed by performing a melt curve analysis at the end of each PCR run.

The absolute target copy number for each gene studied was then normalized using the HPRT gene as the reference ‘housekeeping’ gene, and the result expressed as the absolute copy number of target gene mRNA per 10^5^ absolute HPRT copy number. When the result was expressed as a percentage change against control, the absolute target gene number for both genes was first normalized using the HPRT reference gene copy number and the percentage change then calculated.

### Statistics

Graphpad Prism software and a two-tailed Mann–Whitney test were used for data analysis.

## Author contributions

SS and IT conceived and designed studies, synthesized compounds, performed biological studies and wrote the paper. TSB and MZ performed modelling studies. TB, KAJ, PMR and SMT synthesized and analyzed dendrimers. PeS and SM analyzed PETIM-DG by NMR. BM, AP, PJS and CT made *Shigella* mutants and performed animal studies. IT and PaS designed RT-PCR primer pairs, and IT designed and constructed quantification plasmids and performed RT-PCR studies. All authors revised and approved the manuscript.

## Supplementary material

Detailed facts of importance to specialist readers are published as ”Supporting Information”. Such documents are peer-reviewed, but not copy-edited or typeset. They are made available as submitted by the authors.
