# Supplementary material for: Preventing acute gut wall damage in infectious diarrhoeas with glycosylated dendrimers
Source: EMBO Mol Med. 2012 Aug 6;4(9):866–81. doi: 10.1002/emmm.201201290 (PMC3491821; doi:10.1002/emmm.201201290)
Supplement: Supplementary file 1 [file emmm0004-0866-SD1.pdf]

EMBO
Molecular Medicine

Manuscript EMM-2012-01290

Preventing acute gut wall damage in infectious diarrhoeas with glycosylated dendrimers

Ian Teo, Steve M. Toms, Benoit Marteyn, Teresa S. Barata, Peter Simpson, Karen A. Johnston, Pamela Schnupf, Andrea Puhar, Tracey Bell, Chris Tang, Mire Zloh, Steve Matthews, Phillip M. Rendle, Philippe J. Sansonetti, and Sunil Shaunak

Corresponding author: Sunil Shaunak, Imperial College London

Review timeline:

	Submission date:	15 February 2012
	Accepted:	22 June 2012

Transaction Report:

No Peer Review Process File is available with this article, as the authors have chosen not to make the review process public in this case.
