# Supplementary material for: Preventing acute gut wall damage in infectious diarrhoeas with glycosylated dendrimers
Source: EMBO Mol Med. 2012 Aug 6;4(9):866–81. doi: 10.1002/emmm.201201290 (PMC3491821; doi:10.1002/emmm.201201290)
Supplement: Supplementary file 2 [file emmm0004-0866-SD2.pdf]

Preventing acute gut wall damage in infectious diarrhoeas with glycosylated dendrimers

Teo et al.

Supplementary Data set listing

Figure S1A:
Capillary electrophoresis of G3.5 PAMAM-$(COONa)_{64}$ dendrimer at 214 nm using positive polarity with $LiOH/H_3BO_3$ buffer at pH 9.1 and a capillary voltage of 30 kV.

Figure S1B:
Capillary electrophoresis of G3.5 PAMAM-DG at 214 nm using positive polarity with $LiOH/H_3BO_3$ buffer at pH 9.1 and a capillary voltage of 30 kV.

Figure S2:
PAMAM-DG had no antibacterial activity against the Gram-negative Enterobacteriaceae *Escherichia coli* at 5 mg/ml.

Figure S3:
Neither the 6'-sulfated nor the 6'-phosphorylated PAMAM-DG had any effect on LPS-induced cytokine responses in human monocytes.

Figure S4:
PAMAM-DG had no antibacterial activity against the Gram-negative Enterobacteriaceae *Shigella flexneri* at 7 mg/ml.

Figure S5:
^{1}H NMR spectrum (600 MHz). PETIM-$(COOH)_{16}$.14HCl dendrimer and
PETIM-DG {1,7-diamino-4-oxaheptyl[*N*,*N*,*N*',*N*']:{8-aza-4-oxaoctyl(8,8)}$^{G1,G2}_{4x,8x}$:
(2-carboxyethyl)$_{12}$(3-glucosamino-3-oxopropyl)$_{4}$-cascadane}.

Figure S6:
Size exclusion chromatography of PETIM-DG using a Superdex peptide exclusion column.

Figure S7A:
PETIM-DG:- HPLC-CAD of fraction B of PETIM-DG from Figure 3G in the paper.

Figure S7B:
PETIM-DG:- ^{1}H-NMR spectrum of fraction B of PETIM-DG from Figure 3G in the paper.
^{1}H-NMR: (500 MHz, D_2O, 303K): δ 5.28–5.26 (m, 3H), 3.99–3.18 (m, 225H), 2.97–2.93 (m, 30H), 2.76–2.71 (m, 15H), 2.18–2.05 (m, 69H), 1.27–1.22 (m, 9H).

Figure S8A:
PETIM-DG:- HPLC-CAD of fraction D of PETIM-DG from Figure 3G in the paper.

Figure S8B:
PETIM-DG:- ^{1}H-NMR spectrum of fraction D of PETIM-DG from Figure 3G in the paper.
^{1}H-NMR: (500 MHz, D_2O, 303 K): δ 5.28–5.25 (m, 3H), 3.98–3.18 (m, 228H), 2.96–2.92 (m, 30H), 2.83–2.75 (m, 12H), 2.19–2.05 (m, 69H), 1.27–1.22 (m, 12H).

Figure S9A:
Stability studies - A:
(i) PETIM-DG was bioactive in human monocytes at 200 µg/ml after storage at 4ºC for 9 months.

(ii) PETIM-$(COOH)_{16}$ dendrimer (PETIM-D) did not block LPS-induced cytokine responses.

Figure S9B:
Stability studies - B:
(i) PETIM-DG was bioactive in human monocytes at 200 µg/ml after storage in sealed vials in a humidified 37^0C incubator for 42 days (n = 3).

(ii) PETIM-DG in sealed vials was heated to 70ºC for 1 h. It remained bioactive at 200 µg/ml (n = 3).

(iii) ^{1}H-NMR: (500 MHz, D_2O, 303 K) of PETIM-DG in sealed vials after heating to 70ºC for 1 h.

Figure S10:
PETIM-DG at 600 µg/ml was not toxic to cells in whole human blood when cultured at 37°C for 48 h and analyzed by Coulter flow cytometry for erythrocytes, neutrophils, lymphocytes, monocytes, eosinophils, basophils and platelets.

Figure S11:
In intestinal epithelial Caco-2 cells which are functionally intact for both TLR4 and intracellular LPS signaling but without MD-2, neither LPS nor PETIM-DG had an effect on cytokine responses.

Figure S12:
PETIM-DG had no antibacterial activity against *E. coli* at 5 mg/ml.

Figure S13:
Treatment with intraperitoneal PAMAM-DG at 20 mg/kg was associated with a reduction in IL-6 and TNF-α mRNA expression in Peyer’s patches.

Figure S14:
PAMAM-DG had no *in vivo* antibacterial effect against *Shigella flexneri* in the rabbit ligated ileal loop model.

Figure S15:
Shigella flexneri (Sf) IcsA mRNA expression (an indicator of bacterial gut wall invasion) was reduced by 82 ± 8% by PAMAM-DG at 5 mg/loop at 12 h.

Figure S16:
Gut tissue immuno-histochemistry for *Shigella* LPS confirmed a large reduction in bacterial gut wall invasion in rabbits treated with PAMAM-DG.

Table S1:
Human, rabbit and shigella primers pairs for quantitative real-time RT-PCR studies.

Figure S1A:

Capillary electrophoresis of G3.5 PAMAM-$(COONa)_{64}$ dendrimer at 214 nm
using positive polarity with $LiOH/H_3BO_3$ buffer at pH 9.1 and a capillary voltage of 30 kV

Chromatograms
aligned using
the water (w) peak.

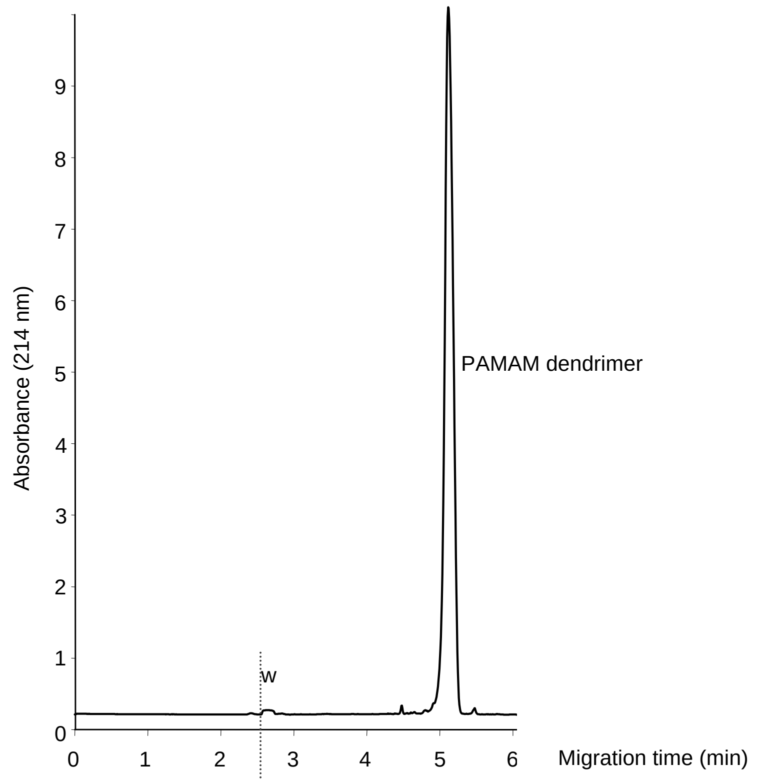

Figure S1B:

Capillary electrophoresis of G3.5 PAMAM-DG at 214 nm
using positive polarity with $LiOH/H_3BO_3$ buffer at pH 9.1 and a capillary voltage of 30 kV

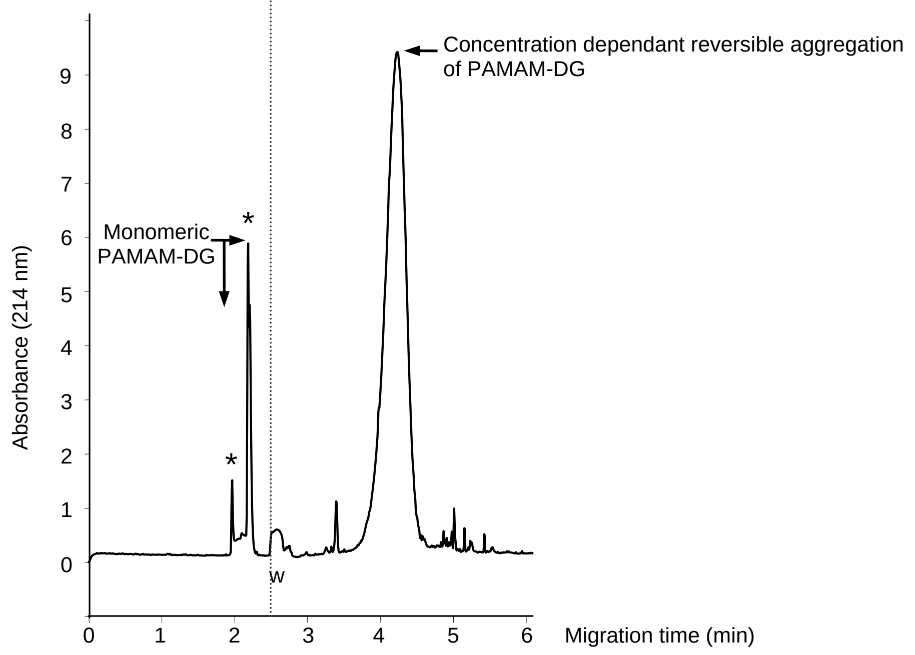

* Monomeric PAMAM-DG. The two peaks represent α/β mutarotation of the anomeric C1 carbon of the attached glucosamines. Their relative ratio is concentration, temperature and pH dependent.

Figure S2:

<u>PAMAM-DG had no antibacterial activity against the</u>
<u>Gram-negative Enterobacteriaceae *Escherichia coli* at 5 mg/ml</u>

E. coli in log phase growth was diluted in Terrific broth to give an optical density of 0.1 units/ml at 600 nm. One hundred μl of PAMAM-DG at 50 mg/ml (dissolved in water) was added to each ml of the *E. coli* culture to give a final PAMAM-DG concentration of 5 mg/ml. The same volume of water was added to the control *E. coli* culture. Optical densities of the bacterial cultures were read in triplicate at 30 min intervals during the following 4 hours of logarithmic bacterial growth. There was no change in the rate of growth of *E. coli* in the presence of PAMAM-DG.

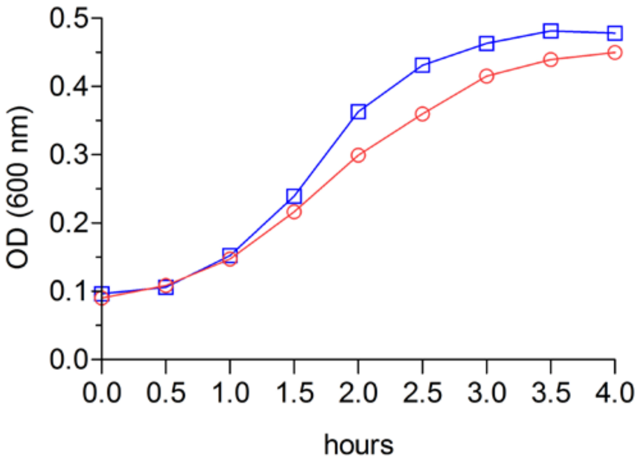

Blue = Control *E. coli*.
Red = *E. coli* + PAMAM-DG at 5 mg/ml.

Pooled data (n = 3) shown as mean ± sem.

Figure S3:

<u>Neither the 6'-sulfated nor the 6'-phosphorylated PAMAM-DG
had any effect on LPS-induced cytokine responses in human monocytes</u>

Salmonella minnesota LPS was used at 25 ng/ml. All compounds were tested at 200 µg/ml. The results are shown for IL-6 and TNF-α.

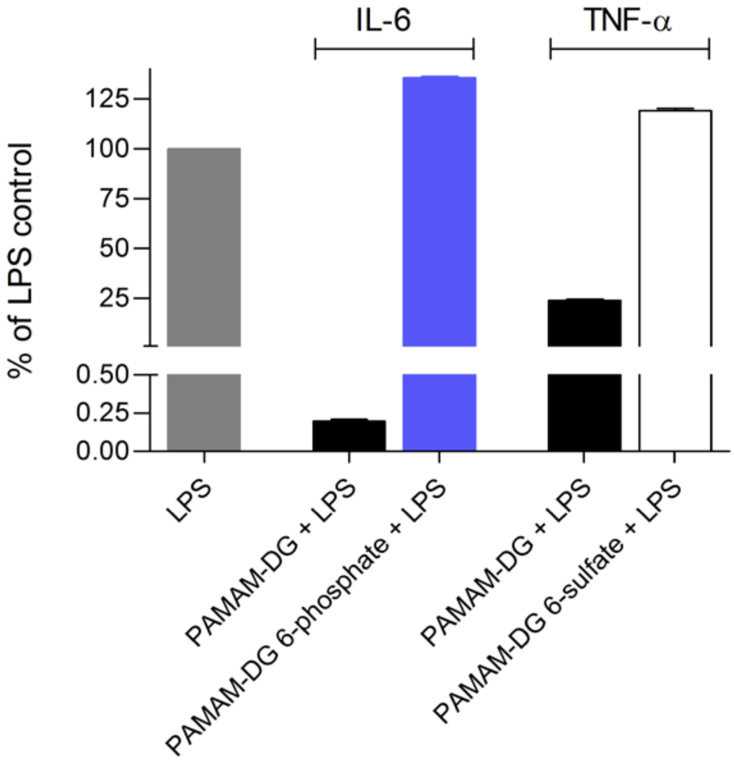

Pooled data (n = 3) shown as mean ± sem.

Figure S4:

<u>PAMAM-DG had no antibacterial activity against</u>
<u>the Gram-negative Enterobacteriaceae *Shigella flexneri* at 7 mg/ml</u>

Shigella flexneri were diluted in growth media to a titre of ~0.5 x 10^6 colony forming units/ml. Aliquots were then treated with PAMAM-DG at 7 mg/ml. At time zero, a 10 µl aliquot of each culture was removed, diluted and plated onto Congo red plates to determine the number of colony forming units (CFU). PAMAM-DG treated and control cultures were then grown at 37^0C with shaking for 8 h and plated on Congo red plates to determine the number of CFUs.

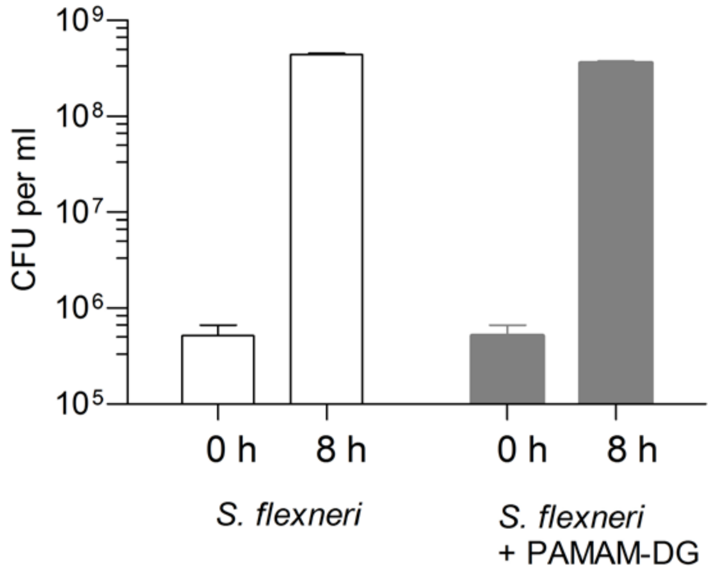

Time zero titre of *S. flexneri* = 5.2 $\underline{+}$ 1.4 x 10^5 CFUs.
8 hour titre of *S. flexneri* = 4.5 $\underline{+}$ 0.15 x 10^8 CFUs.
8 hour titre of *S. flexneri* + PAMAM-DG (7 mg/ml) = 3.4 $\underline{+}$ 0.16 x $10^{\mathbf{8}}$ CFUs.

Pooled data (n = 9) shown as mean ± sem on a $\log_{10}$ scale.

Figure S5: ^{1}H NMR spectrum (600 MHz):
PETIM-(COOH)$_{16}$.14HCl dendrimer & PETIM-DG {1,7-diamino-4-oxaheptyl[*N*,*N*,*N*’,*N*’]:{8-aza-4-oxaoctyl(8,8)}$^{G1,G2}_{4x,8x}$:(2-carboxyethyl)$_{12}$(3-glucosamino-3-oxopropyl)$_{4}$-cascadane}.

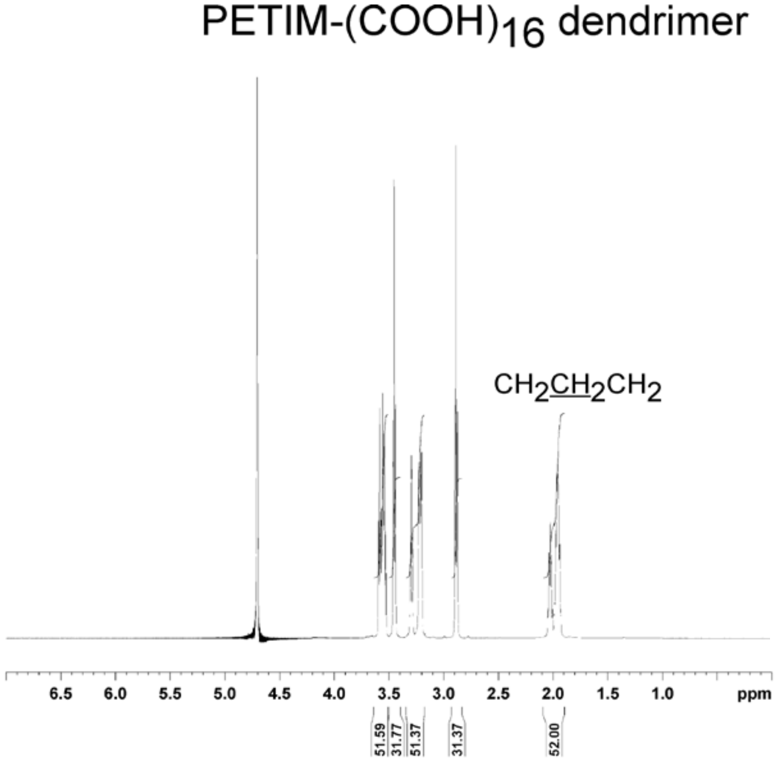

Integrals calibrated to the 52Hs of $CH_2\underline{CH_2}CH_2$

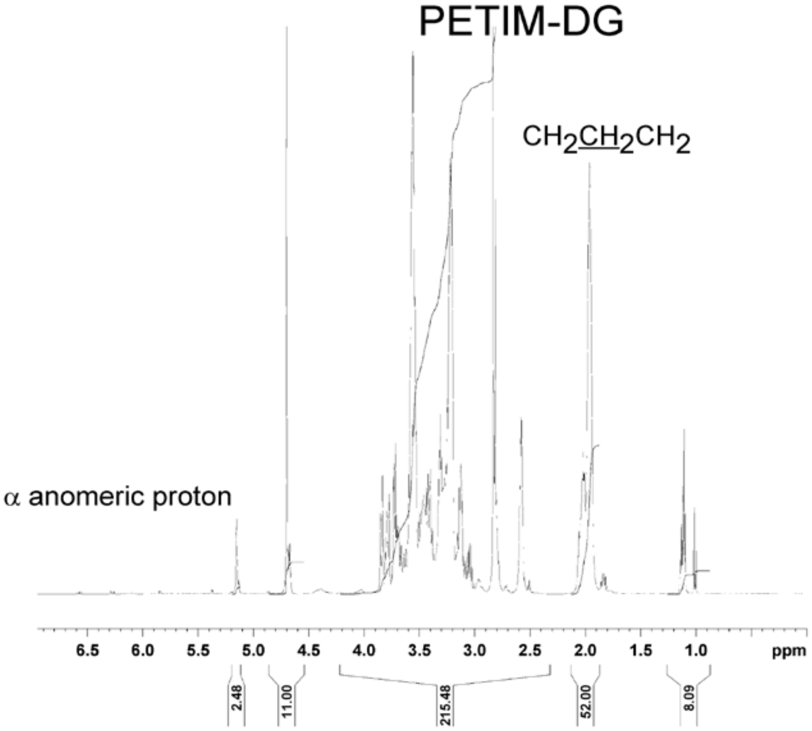

Integrals calibrated to the 52Hs of $CH_2\underline{CH_2}CH_2$
and α:β anomeric ratio measured from ^{1}H-^{13}C HSQC spectrum

Figure S6:

Size exclusion chromatography of PETIM-DG using a Superdex peptide exclusion column

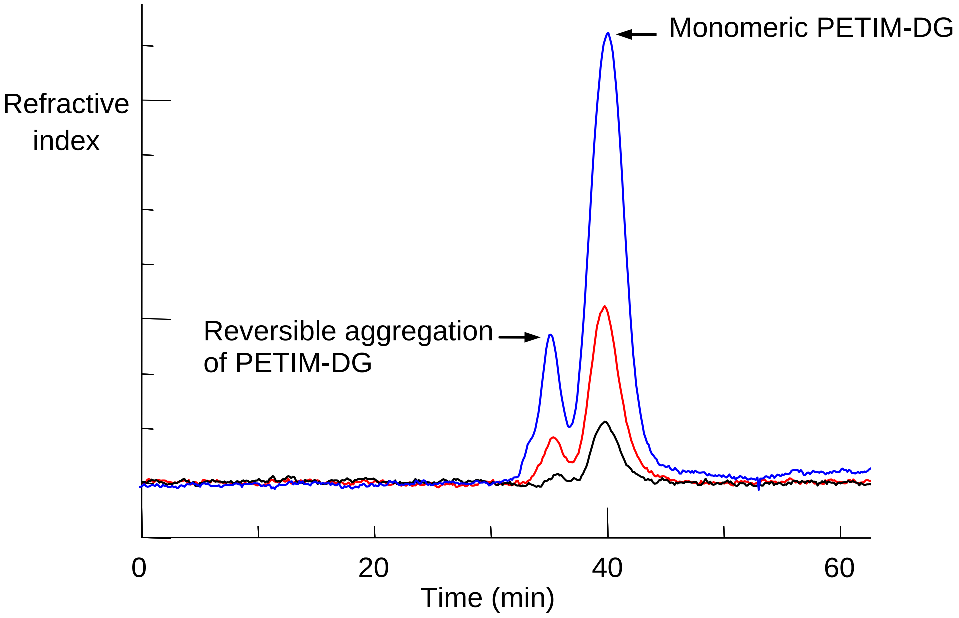

Column loadings of:-
3 mg PETIM-DG
1 mg PETIM-DG
250 µg PETIM-DG

The relative proportion of reversibly aggregated PETIM-DG decreases substantially as the column loading of PETIM-DG is decreased from 3 mg/100 µl to 250 µg/100 µl.

Additional capillary electrophoresis method references for the α and β anomers of glucosamine that relate to Supplementary Figures S1 and S6:

(1) Skelley AM and Mathies RA (2006) Rapid on-column analysis of glucosamine and its mutarotation by microchip capillary electrophoresis. J Chromatography A 1132; 304-309

(2) Hinterwirth H, Lammerhofer M, Preinerstorfer B, Gargano A, Reischl R, Bicker W, Trapp O, Brecker L, Lindner W (2010) Selectivity issues in targeted metabolomics: Separation of phosphorylated carbohydrate isomers by mixed-mode hydrophilic interaction/weak anion exchange chromatography. J Sep Sci 33; 3273-3282

(3) Pazourek J (2010) Monitoring of mutarotation of monosaccharides by hydrophilic interaction chromatography. J Sep Sci 33; 974-981

Fraction B of PETIM-DG from Figure 3G in the paper

Figure S7A: HPLC-CAD:

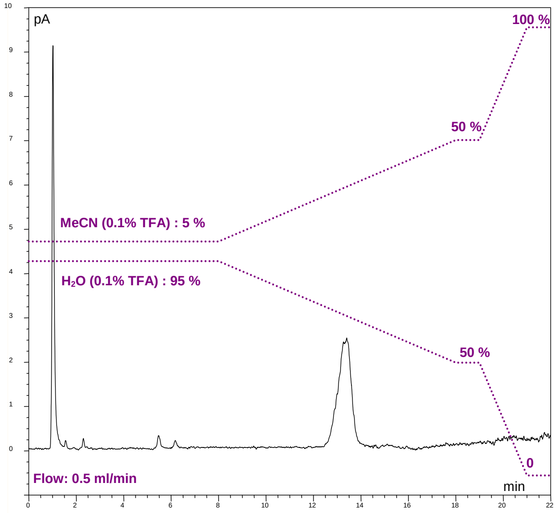

Figure S7B: ^{1}H-NMR:

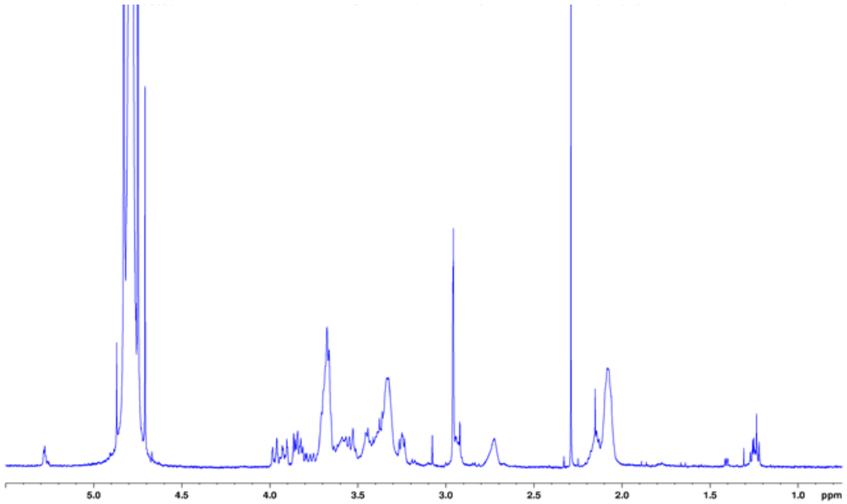

Fraction D of PETIM-DG from Figure 3G in the paper

Figure S8A: HPLC-CAD:

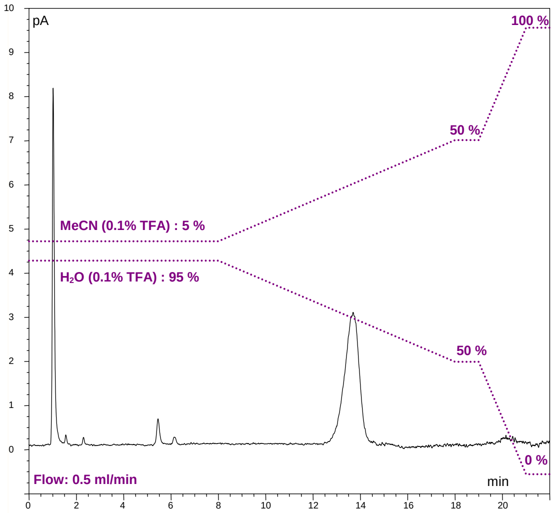

Figure S8B: ^{1}H-NMR:

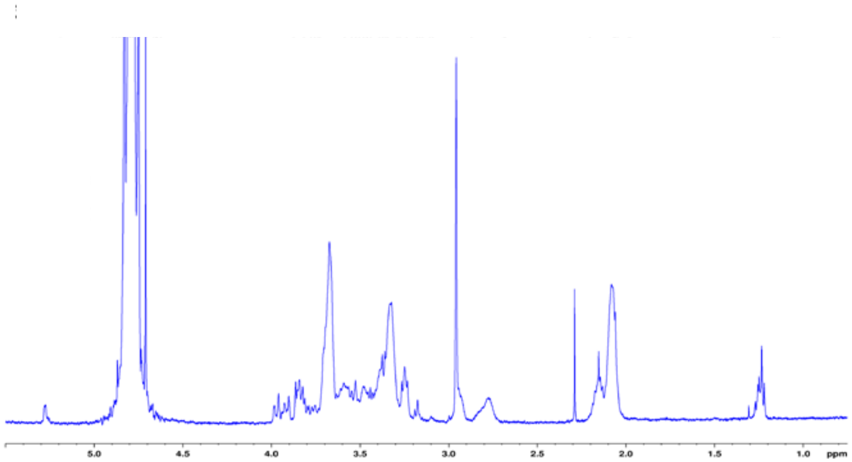

Figure S9A: Stability studies - A

PETIM-DG was bioactive in human monocytes at 200 µg/ml after storage at 4ºC for 9 months.

PETIM-$(COOH)_{16}$ dendrimer (PETIM-D) did not block LPS-induced cytokine responses.

Pooled data (n = 6) shown as mean ± sem on a log_{10} scale.

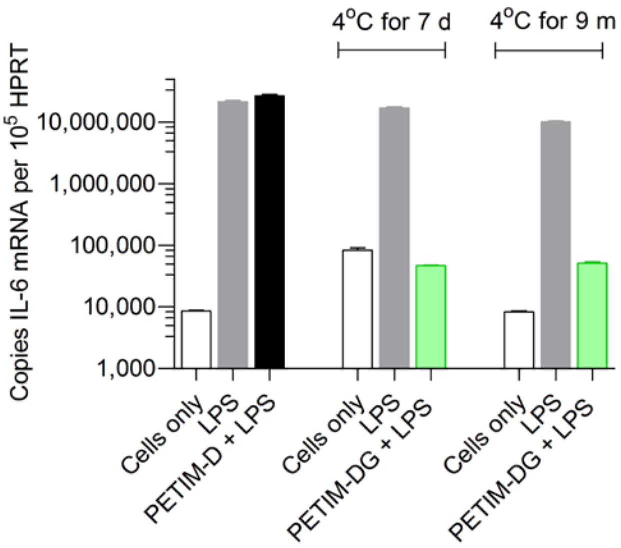

Figure S9B: Stability studies - B

PETIM-DG was bioactive in human monocytes at 200 µg/ml after storage in sealed vials in a 100% humidified 37^{0}C bioincubator for 42 days.

PETIM-DG in sealed vials was heated to 70ºC for 1 h. It remained bioactive at 200 µg/ml.

Pooled data (n = 3) shown as mean ± sem on a log_{10} scale.

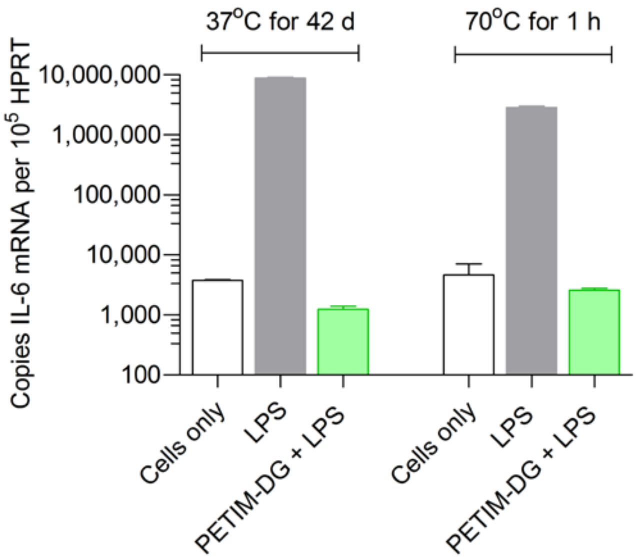

^{1}H-NMR of PETIM-DG after heating to 70ºC for 1 h:
It has not changed when compared to the ^{1}H-NMR's shown in Supplementary Figures S7B & S8B:

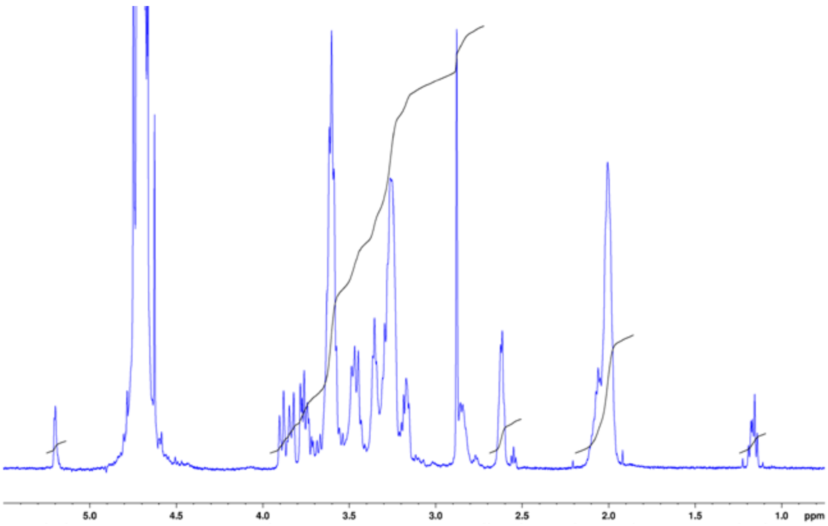

Figure S10:

PETIM-DG at 600 μg/ml was not toxic to cells in whole human blood when cultured at 37°C for 48 h and analyzed by Coulter flow cytometry for erythrocytes, neutrophils, lymphocytes, monocytes, eosinophils, basophils and platelets.

WBC	1.64	-	[10^9/L]			
RBC	2.51		[10^12/L]			
HGB	8.2		[g/dL]			
HCT	0.302		[Ratio]			
MCV	120.3	+	[fL]			
MCH	32.7		[pg]			
MCHC	27.2	-	[g/dL]			
PLT	89		[10^9/L]			
RDW-SD	74.9	+	[fL]			
RDW-CV	17.4	+	[%]			
PDW	9.4		[fL]			
MPV	8.9	-	[fL]			
P-LCR	16.9		[%]			
PCT	0.08	-	[%]			
NEUT	1.06	-	[10^9/L]	64.7		[%]
LYMPH	0.42	-	[10^9/L]	25.6		[%]
MONO	0.13		[10^9/L]	7.9		[%]
EO	0.03		[10^9/L]	1.8		[%]
BASO	0.00		[10^9/L]	0.0		[%]
NRBC	0.00		[10^9/L]	0.0		[/100WBC]
RET	0.78		[%]	19.6		[10^9/L]
IRF	3.0		[%]			
LFR	97.0		[%]			
MFR	3.0		[%]			
HFR	0.0		[%]			

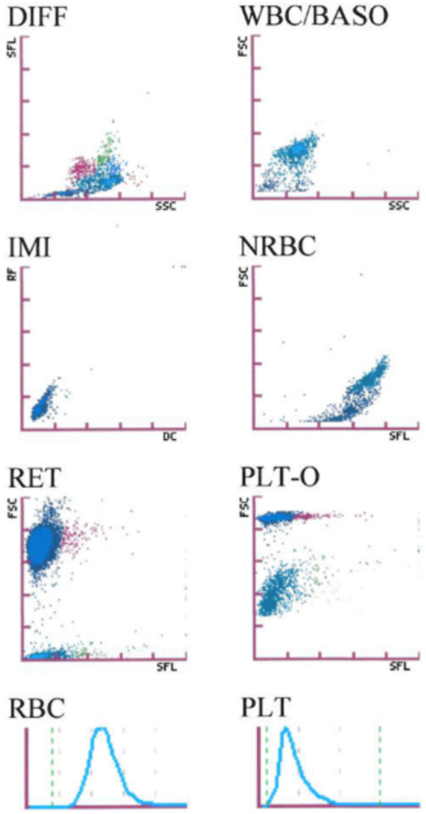

Whole human blood control cultured at 37°C for 48 h.

Results from one of four representative experiments shown.

WBC	1.67	-	[10^9/L]			
RBC	2.52		[10^12/L]			
HGB	8.2		[g/dL]			
HCT	0.303		[Ratio]			
MCV	120.2	+	[fL]			
MCH	32.5		[pg]			
MCHC	27.1	-	[g/dL]			
PLT	100		[10^9/L]			
RDW-SD	75.7	+	[fL]			
RDW-CV	17.6	+	[%]			
PDW	14.2		[fL]			
MPV	11.0		[fL]			
P-LCR	34.1		[%]			
PCT	0.11	-	[%]			
NEUT	1.26	-	[10^9/L]	75.4	+	[%]
LYMPH	0.30	-	[10^9/L]	18.0	-	[%]
MONO	0.09		[10^9/L]	5.4		[%]
EO	0.02		[10^9/L]	1.2		[%]
BASO	0.00		[10^9/L]	0.0		[%]
NRBC	0.00		[10^9/L]	0.0		[/100WBC]
RET	0.83		[%]	20.9		[10^9/L]
IRF	4.3		[%]			
LFR	95.7		[%]			
MFR	3.8		[%]			
HFR	0.5		[%]			

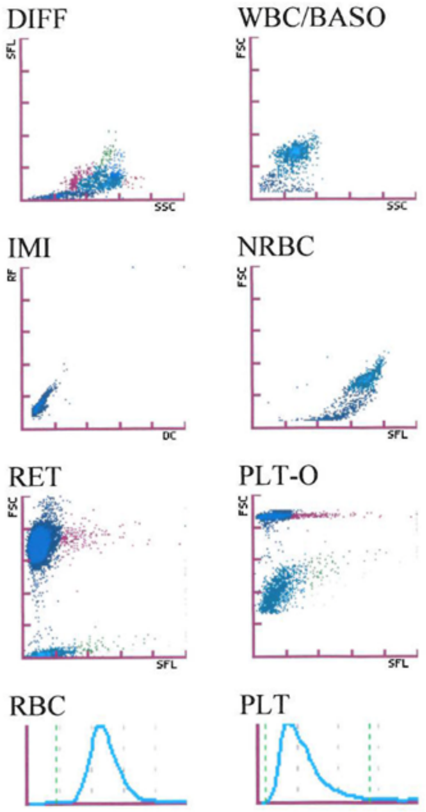

Whole human blood cultured at 37°C with PETIM-DG at 600 μg/ml for 48 h.

Figure S11:

<u>In intestinal epithelial Caco-2 cells which are functionally intact for both TLR4 and intracellular LPS signaling but without MD-2, neither LPS nor PETIM-DG had an effect on cytokine responses</u>

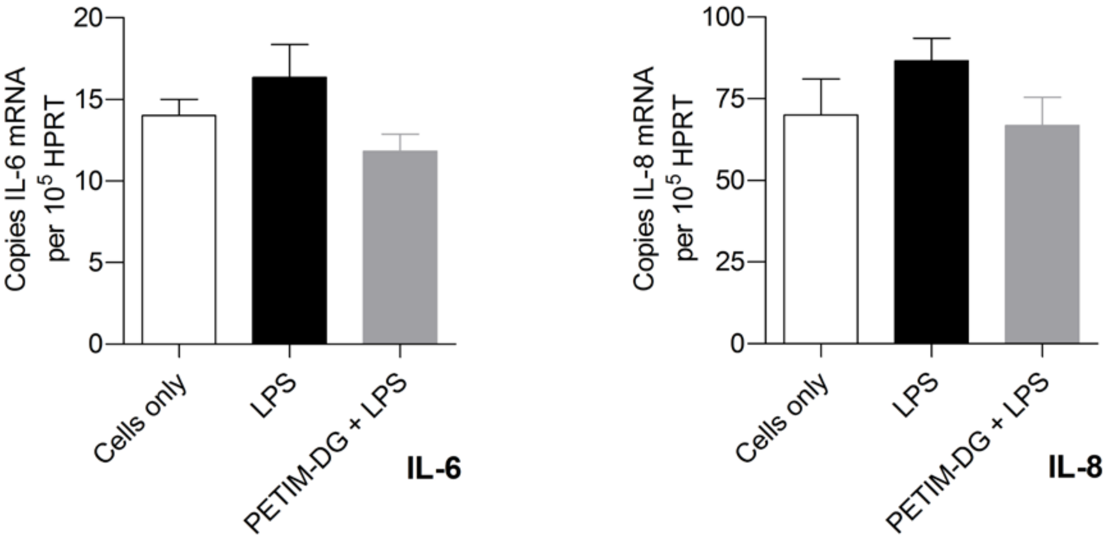

Pooled data (n = 3) shown as mean ± sem.

Figure S12:

<u>PETIM-DG had no antibacterial activity against *E. coli* at 5 mg/ml</u>

E. coli in log phase growth was diluted in Terrific broth to give an optical density of 0.1 units/ml at 600 nm. One hundred µl of PETIM-DG at 50 mg/ml (dissolved in water) was added to each ml of the *E. coli* culture to give a final PETIM-DG concentration of 5 mg/ml. The same volume of water was added to the control *E. coli* culture. Optical densities of the bacterial cultures were read in triplicate at 30 min intervals during the following 4 hours of logarithmic bacterial growth. There was no change in the rate of growth of *E.coli* in the presence of PETIM-DG.

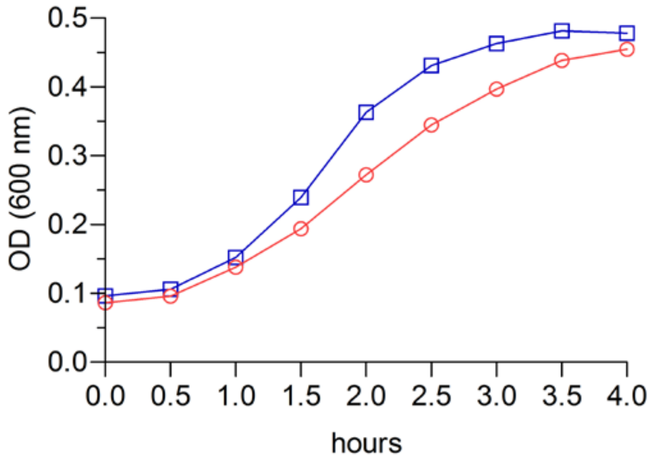

Blue = Control *E. coli*.
Red = *E. coli* + PETIM-DG at 5 mg/ml.

Pooled data (n = 3) shown as mean ± sem.

Figure S13:

<u>Treatment with intraperitoneal PAMAM-DG at 20 mg/kg was associated with a reduction in IL-6 and TNF-α mRNA expression in Peyer's patches</u>

IL-6 and TNF-α mRNA expression were reduced by 65% and 52% respectively compared to the infected untreated control (left-hand panel).

For comparison, the results from Figures 6A & C in the main text of the paper are also shown in which PAMAM-DG (5 mg/loop) was given directly into the ligated ileal loop. IL-6 and TNF-α mRNA expression were reduced by >99.9% and 84% respectively compared to the infected untreated control (right hand panel).

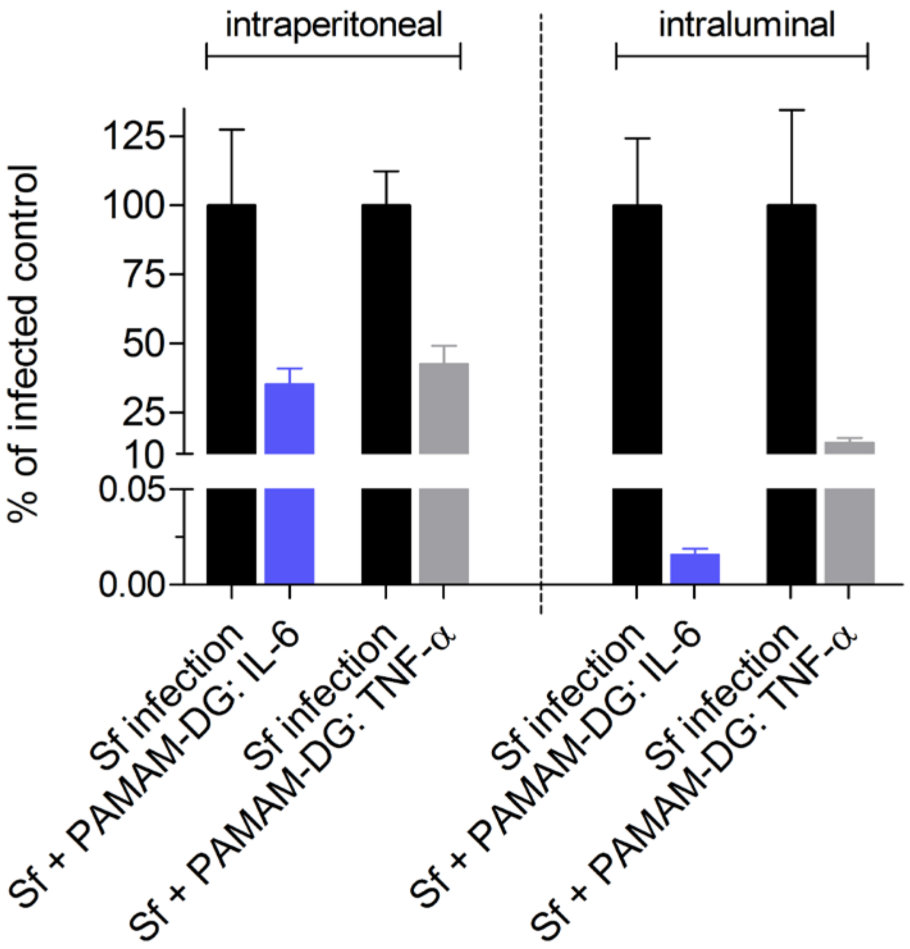

For the intraperitoneal treatment, pooled data (n = 3) shown as mean ± sem.
For the intraluminal treatment, pooled data (n = 4) shown as mean ± sem.

Figure S14:

PAMAM-DG had no *in vivo* antibacterial activity in the rabbit ligated ileal loop model

At the end of the experiment, the intraluminal fluid in the loops was collected and the CFU of *Shigella flexneri* determined using Congo Red plates. The total CFUs/infected ileal loop was determined by multiplying the CFU by the volume of fluid in each loop. The infecting innoculum of *Shigella flexneri* was 10^7 organisms.

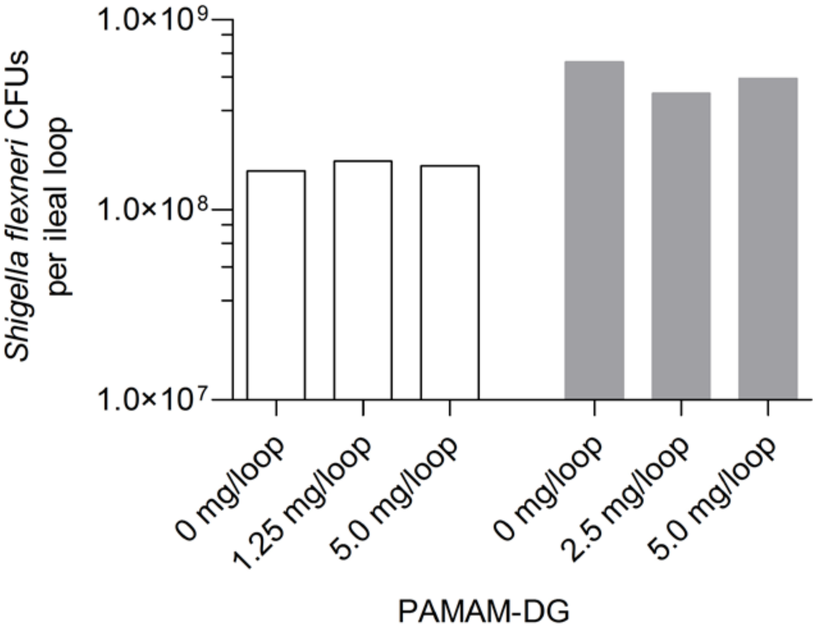

The white bars are from one rabbit experiment and the grey bars are from a second rabbit experiment.

Figure S15:

<u>*Shigella flexneri* IcsA mRNA expression (an indicator of bacterial gut wall invasion) was reduced by 82 ± 8% by PAMAM-DG at 5 mg/loop at 12 h</u>

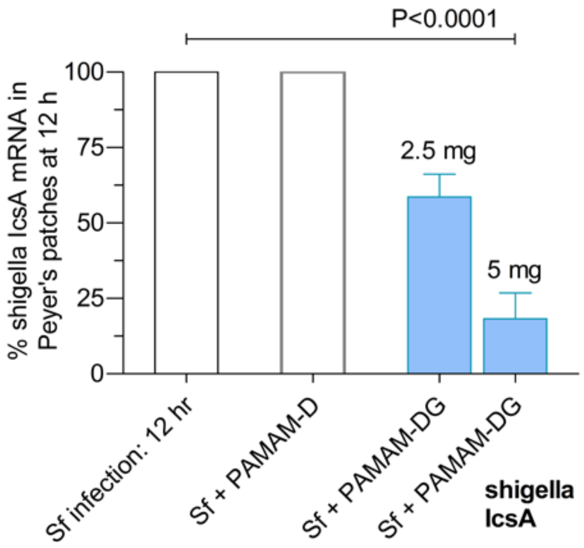

Sf = *Shigella flexneri*
PAMAM-D is PAMAM dendrimer.

Pooled data (n = 3) shown as mean ± sem on a $\log_{10}$ scale.
P value determined using a two-tailed Mann-Whitney test.

Figure S16:

Gut tissue immuno-histochemistry for *Shigella* LPS confirmed
a large reduction in bacterial gut wall invasion in rabbits treated with PAMAM-DG

Tissue sections of Peyer's patches were prepared and immuno-histochemically stained for *Shigella* (brown) with a murine polyclonal anti-*Shigella flexneri* 5a LPS serum and counterstained with hematoxylin as previously described (Boullier et al., 2009; Schnupf et al., 2012). Representative images are shown. The left hand panel shows the brown immuno-histochemical staining for *Shigella* LPS in a Peyer's patch from an infected untreated rabbit. There is a large amount of brown staining in the Peyer's patch whose surface epithelium has been destroyed by the infection. The right hand panel shows the immuno-histochemical staining for *Shigella* LPS in a Peyer's patch from an infected and PAMAM-DG (5 mg/loop) treated rabbit. There is very little brown staining in the Peyer's patch whose surface epithelium remains intact. Results from one of three representative experiments shown.

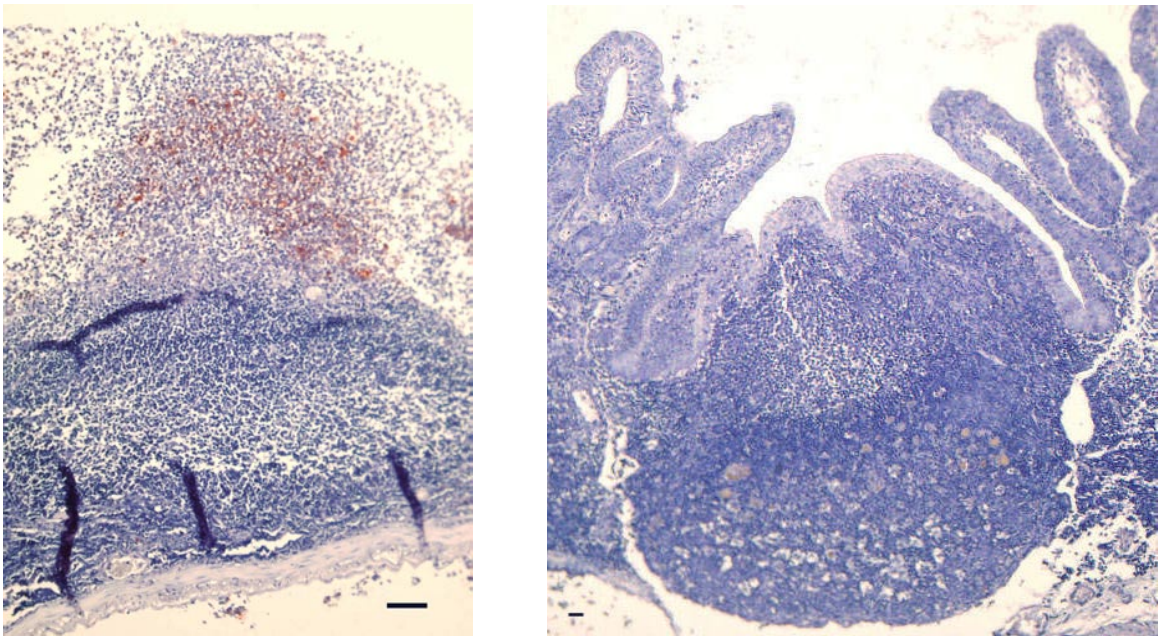

Infected untreated rabbit — Infected and PAMAM-DG treated rabbit

References:

Boullier S, Tanguy M, Kadaoui KA, Caubet C, Sansonetti P, Corthe´sy B, Phalipon A (2009) Secretory IgA-mediated neutralization of Shigella flexneri prevents intestinal tissue destruction by down-regulating inflammatory circuits. J Immunol 183: 5879-5885

Schnupf P, Sansonetti PJ. Quantitative RT-PCR profiling of the rabbit immune response: assessment of acute *Shigella flexneri* infection (2012) PLoS One 7:e36446. doi:10.1371/journal.pone.0036446

Table S1: Human, rabbit and Shigella primers pairs for quantitative real-time RT-PCR studies:

Human (h) gene	*5'-3' sequence of primer pairs*	*Tm (^0C)*	*Length*
hIL-6 (f)	CACACAGACAGC CACTCACCTC	67.3	136
(r)	CTGCCAGTGCCTCTTTGCTG	68.4	
hIL-8 (CXCL-8) (f)	GCGCCAACACAGAAATTATTGTAA	66.3	121
(r)	TTATGAATTCTCAGCCCTCTTCAA	65.6	
hTNF-α (f)	AGGCGGTGCTTGTTCCTCA	68.2	165
(r)	GTTCGAGAAGATGATCTGACTGCC	67.2	
hIL-1β (f)	CCCACAGACCTTCCAGGAGA	66.6	138
(r)	CGGAGCGTGCAGTTCAGTG	68.4	
hCCL3 (MIP-1α) (f)	TGCTGCTTCAGCTACACCTC	63.2	159
(r)	GCACTCGGCTCCAGGTCACT	69.2	
hCCL4 (MIP-1β) (f)	ACCCTCCCACCGCCTGCTGC	76.9	190
(r)	GTTCCAGGTCATACACGTACTCC	63.4	
hIL-10 (f)	CTGAGAACCAAGACCCAGACAT	66	124
(r)	CACGGCCTTGCTCTTGTTT	65.2	
hIFN-β1 (f)	TGCTCTCCTGTTGTGCTTC	66.6	222
(r)	CATCTCATAGATGGTCAATGCG	67.1	
hIFN-γ (f)	AAACGAGATGACTTCGAAAAGCTGA	67.8	111
(r)	ACAACCATTACTGGGATGCTCTTC	66	
hHPRT (f)	GCTCGAGATGTGATGAAGGAG	63.9	190
(r)	TCCCCTGTTGACTGGTCATT	64.4	

Rabbit (r)gene	*5'-3' sequence of primer pairs*	*Tm (^0C)*	*Length*
rIL-6 (f)	CTACCGCTTTCCCCACTTCAG	66.8	113
(r)	TCCTCAGCTCCTTGATGGTCTC	66.8	
rIL-8 (CXCL-8) (f)	GTGCAAATTCAGAAATCATTGTAAA	62.7	125
(r)	TTATGACTCTTGCTGCTCAGCCCTCTTCAA	74.7	
rTNF-α (f)	CTGCACTTCAGGGTGATCG	64.6	132
(r)	CTACGTGGGCTAGAGGCTTG	63.7	
rIL-10 (f)	GAACAGCTGCATTCACTTTCCAG	66.9	235
(r)	TGATGGCTGGACTGTGGTTC	66.3	
rCCL4 (MIP-1β) (f)	TCTCGTACACCCTGCGGAAGCTT	71.7	164
(r)	GTTCCAAGTCATCCACGTACTCCT	66.2	

rIL-1β (f)	TTGAAGAAGAACCCGTCCTCTG	66.5	128
(r)	CTCATACGTGCCAGACAACACC	66.6	
rIFN-β1 (f)	AATCGCTCTCCTGTTGTGCTTC	66.6	132
(r)	GCAGTCCTCAGTCGTTCCATTC	67.1	
rIFN-γ (f)	TGCCAGGACACACTAACCAGAG	66.4	126
(r)	TGTCACTCTCCTCTTTCCAATTCC	66.4	
Defensin b 103 (f)	ATGAGGATCCATTATCTCCTGTTTGC	65.9	155
(r)	ATCTGTTCCTCCTTTGGAAGGCAGCT	72.1	
CD3 Delta chain (f)	CTGGACATGAGACTGGAAGGCT	66.8	139
CD3 Delta chain (r)	TCACTTGTTCCGAGCCCAGTT	68.0	
Fox P3 (f)	CAACATGGACTACTTCAAGTTCC	61.4	182
Fox P3 (r)	TGGCGGATGGCGTTCTTCCAGGT	77.8	
TGF-β (f)	GCTGTACATTGACTTCCGCA	63.9	219
TGF-β (r)	CACGTAGTACACGATGGGCA	64.6	
Caspase-3 (f)	AATGGATTATCCTGAAATGGG	61.3	225
(r)	CTGCTCCTTTTGCTATGATCTTC (annealing at 56^{0}C)	63.0	
rHPRT (f)	GCTCGAGATGTGATGAAGGAGA	65.6	190
(r)	TCCCCTGTTGACTGGTCATTAC	64.9	

Shigella gene	*5'-3' sequence of primer pairs*	*Tm (^{0}C)*	*Length*
IcsA (f)	TGATGGTGGTGAGGCTGTTA	64.2	263
IcsA (r)	CGAACGTGCCCTTATTGATT	63.6	
